# Multidimensional Grocery-Store Preferences of Low-Income Caregivers of Toddlers

**DOI:** 10.1016/j.cdnut.2026.109530

**Published:** 2026-09-12

**Authors:** Aihui Li, Xingguo Wang, Stephanie Rogus, Yu Yvette Zhang, Alexandra L MacMillan Uribe

**Affiliations:** 1 Department of Agricultural Economics, Texas A&M University, College Station, TX, United States; 2 Department of Agricultural Economics, Texas A&M University, Fort Worth, TX, United States; 3 Texas A&M AgriLife Research at El Paso, El Paso, TX, United States; 4 Institute for Advancing Health Through Agriculture, Texas A&M AgriLife Research, Dallas, TX, United States; 5 Department of Nutrition, College of Agriculture and Life Sciences, Texas A&M University, College Station, TX, United States

**Keywords:** grocery store preferences, low-income caregivers, toddlers, factor analysis, multivariate regression, food access

## Abstract

**Background:**

Many United States toddlers do not meet recommended fruit and vegetable intakes, and intakes are lower in low-income households. Where caregivers shop shapes what they buy, yet studies of store choice have generally examined price and proximity alone, leaving other features of the retail environment largely unexamined.

**Objectives:**

We examined how household characteristics influence grocery-store preferences among caregivers of toddlers from low-income households in Texas.

**Methods:**

We conducted a cross-sectional survey in which caregivers of toddlers aged 12 to 47 mo from low-income households residing in Dallas and Tarrant Counties, Texas, United States rated 37 store-attribute items. Exploratory factor analysis with oblique rotation identified latent preference dimensions, and factor scores were modeled as dependent variables in multivariable linear regressions. The sample consisted of 148 caregivers with a mothers-only subsample (*n* = 121) analyzed separately.

**Results:**

Five preference dimensions were identified: store quality and service, in-store accessibility, online and delivery options, price sensitivity, and food quality and health. Older caregivers and Hispanic participants showed higher preferences for store quality and service, in-store accessibility, and food quality and health. In mutually adjusted models, frequent food preparation was positively associated with most dimensions and frequent food shopping was negatively associated with several. The 2 indicators agreed for most caregivers, so the contrast between them was identified by 11 discordant caregivers and was estimated imprecisely. Supplemental Nutrition Assistance Program participation corresponded to lower price sensitivity. Among mothers, non-Hispanic Black predicted greater concern for food quality and health, whereas employment was linked to lower price sensitivity.

**Conclusions:**

Grocery-store preferences among low-income caregivers of toddlers reflect multidimensional food-environment considerations that vary systematically by demographic and behavioral factors, with distinctive patterns among mothers. Programs aiming to improve access to fruits and vegetables should consider caregivers’ roles, routines, and participation in food assistance programs when designing interventions.

## Introduction

Healthy food access for parents of toddlers is shaped by a dense web of demographic, economic, and spatial factors. Parents need to navigate a complex retail environment while prioritizing nutrition, safety, convenience, and cost. These needs have intensified as grocery formats diversify. A growing body of literature has documented individual drivers of store choice, such as price sensitivity or geographic proximity [1,2]. Yet, there remains a limited understanding of how multiple household characteristics combine to influence parental decisions when purchasing fresh fruits and vegetables (FVs) for young children. This gap is especially consequential because most United States toddlers do not meet recommended FV intake levels, with intakes notably lower among children in low-income households [3]. The toddler stage is also a period when dietary habits exert long-term influence on health trajectories [4].

The literature consistently identifies price sensitivity and convenience as the twin pillars of grocery-store choice. Lower-income households tend to gravitate toward discounters, private labels, and weekly promotions, whereas higher-income shoppers often trade money for time by frequenting premium outlets or using paid delivery services [5]. Time costs also matter: a 10-min increase in round-trip store distance can reduce shopping frequency by 8% among working parents [2]. Nutrition and food safety concerns further shape purchasing behavior. Adults scoring higher on validated nutrition knowledge indices are substantially more likely to meet the recommended intakes of FVs and fat [6]. Yet, limited budgets and participation in the Supplemental Nutrition Assistance Program (SNAP) or the Special Supplemental Nutrition Program for Women, Infants, and Children (WIC) can narrow the feasible preference set [7].

The expansion of digital channels during the COVID-19 pandemic has added another layer of complexity. A nationally representative Nielsen Consumer Panel survey conducted in July 2020 found that 39% of United States adults had purchased groceries online, and among these users, 89% planned to keep doing so [8]. National data document a durable pivot toward click-and-collect, online ordering paired with curbside pickup, as households continue blending digital and in-store shopping routines [9]. The SNAP Online Purchasing Pilot expanded nationwide during COVID-19, making online grocery purchasing available to SNAP recipients in nearly all states through participating retailers [10]. However, qualitative research among SNAP-eligible households reveals persistent barriers to online produce shopping, including spotty mobile service, delivery fees, and mistrust of produce substitutions [11,12]. For many parents, digital tools supplement rather than substitute for in-person produce inspection.

Despite this extensive body of work, prior studies have typically examined a limited set of store attributes—most commonly price, proximity, or time-related convenience—when analyzing food purchasing behavior. Much less attention has been paid to other features of the retail environment that may shape caregivers’ shopping experiences, such as store cleanliness, safety, cart availability, parking, layout, and overall ease of navigating the store. When these attributes are considered, they are often examined in isolation rather than as part of a broader, interrelated preference structure. As a result, little is known about how traditional determinants of store choice (e.g., cost and convenience) operate together with these less-studied aspects of the store environment in shaping caregivers’ evaluations of FV purchasing locations.

This study addresses this gap by using a behavioral food-environment perspective to examine how demographic, socioeconomic, and caregiving characteristics shape multiple latent dimensions of grocery-store preferences among low-income caregivers of toddlers. Using cross-sectional survey data from 148 caregiver–toddler dyads in the Dallas–Fort Worth metro area, we explore underlying preference structures for FV purchasing and assess how household characteristics relate to each dimension. We further examine whether these relationships differ for mothers, compared with other caregivers, who are typically the primary food purchasers and preparers in households with young children.

By integrating economic, nutritional, technological, and sensory considerations into a unified analytic framework, this study provides a multidimensional view of how modern caregiving contexts shape FV shopping. The focus on low-income families with toddlers recognizes that this group’s purchasing decisions may have disproportionate effects on early-life dietary patterns. The results are intended to generate evidence that can guide retailers in improving store operations and shopping experiences (e.g., service quality, navigability, and online ordering/delivery options) and inform policymakers seeking to refine initiatives like SNAP Online Purchasing to broaden healthy food access for households facing both budget and time constraints. In doing so, the study advances both scholarly understanding and practical strategies to ensure that all families, regardless of income or schedule, can secure affordable, nutritious produce for their young children.

This study makes 3 contributions. The first concerns the population. Low-income caregivers of toddlers are the group that many nutrition assistance programs are designed to reach. FV purchases are a central outcome for those programs. We are not aware of a prior study that describes how these caregivers weigh the attributes of the stores in which those purchases are made. The second concerns the breadth of what is measured. Studies of store choice among low-income households in the United States have generally examined price and proximity alone or have relied on single-item questions asking respondents to name 1 main reason for choosing a store. Attributes such as cleanliness, staff conduct, ease of navigation, online ordering, and delivery have therefore rarely entered the account. We model a broad store-attribute battery jointly and recover 5 dimensions along which these caregivers differ, which allows the trade-offs among store attributes to be read together rather than 1 attribute at a time. The third concerns policy. Food assistance programs reach these households through authorized retailers, and the dimensions recovered here describe features of a store that vendor authorization criteria, which turn on stocking requirements and price, do not address. The battery is adapted from the Food Store Selection Questionnaire of Krukowski et al. [13], whose developers assigned the items to 4 conceptual domains a priori. We identified no subsequent study establishing the instrument’s factor structure or reliability. The dimensions used here are therefore derived from these data rather than imposed a priori. The store-image tradition in the marketing literature is well established, and the novelty of this study lies in applying this multidimensional approach to a previously understudied population and their purchasing decisions.

## Methods

### Study design and sample

Data for the current analysis were obtained from a cross-sectional survey of caregivers of toddlers conducted between March and July 2022. Participants were recruited via research panels managed by Qualtrics, which use quota-based screening to ensure respondents meet all eligibility criteria [14]. This recruitment method also ensures that online surveys are not contaminated by Internet robots, as the survey link is only shared with verified research panel members who meet eligibility criteria. Individuals were eligible if they were a parent or legal guardian aged ≥18 y, had a toddler between 12 and 47 mo, resided in Dallas or Tarrant Counties, TX, and met low-income criteria defined as participation in ≥1 federal food assistance program within the past 12 mo. Dallas and Tarrant Counties contain the cities of Dallas and Fort Worth and together form the core of the Dallas–Fort Worth metropolitan area, which is demographically and economically diverse and where disparities in food access are documented in Dallas [15]. FV intake among young children in Texas is low: among Texas children aged 1 to 5 y in 2021, 29.9% ate no fruit on a given day, and 47.3% ate no vegetables [3]. Caregivers of toddlers in this region are therefore a population for whom the question of where FVs are purchased is consequential. The survey did not field a household income item because income is a sensitive item in survey research, and participation in ≥1 federal food assistance program in the previous 12 mo served as the operational definition of low income. Low-income caregivers who did not participate in such a program were therefore not eligible, and the findings describe low-income caregivers of toddlers who are inside the food assistance system rather than the low-income population as a whole. Toddlers with medical conditions that severely limited eating or drinking were excluded. Qualtrics panel members received standard points-based compensation through the platform for survey completion. This study was approved by the Texas A&M University Institutional Review Board and deemed exempt (IRB2021-1517M).

The survey was completed online via Qualtrics. Before the survey, the consent form was displayed, and by proceeding to the survey, participants indicated their consent to participate in the study. Respondents completed questions covering 3 domains. First, the survey included 38 store-preference measures capturing a wide range of attributes related to store quality and service, in-store accessibility, online and delivery options, pricing, and food quality. These items were later summarized into latent dimensions using factor analysis. These questions were derived from previously validated instruments [13], but were modified to focus specifically on FV preferences. A comprehensive description of the preference items is provided in Table 1. Second, respondents reported FV purchasing and feeding behaviors, including frequency of food shopping and preparation, as well as participation in food assistance programs (e.g., SNAP, WIC, and free or reduced-price school meals). Third, the survey collected demographic and household characteristics, including caregiver age, gender, ethnicity, race, marital status, education level, number of children, daycare attendance frequency, and dinner-out frequency.

**TABLE 1:** Store-preference items included in the survey

Preference	Description
Work	It’s close to my work.
Wipes	The store has disinfecting wipes available.
Variety	I can choose from a wide variety of fruits and vegetables.
Temp	The store is kept at a good temperature.
Spoil	The store has fruits and vegetables that last a long time without spoiling.
Size	I like the size of the store.
Sales	I think the location has good sales.
Safe	I feel safe when I go there.
Respond	The employees at the store respond to my needs.
Quick	I can complete my shopping quickly.
Prices	I think the location has low prices.
Prep	The store has fruits and vegetables that are already prepared (e.g., cut, washed).
Payment	The store has a payment option I like.
Parking	I can easily find parking.
Otherstores	It’s close to other stores.
Organic	I can buy organic/chemical-free produce.
Open	The store is open when I like to shop.
Online	I can order my fruits and vegetables online.
Maintain	The store is well-maintained.
Local	I can buy locally grown fruits and vegetables.
List	I am able to easily find the fruits and vegetables on my list.
Layout	I am familiar with the store’s layout.
Home	It’s close to my home.
Fresh	The fruits and vegetables are fresh.
Familiar	The store has fruits and vegetables I am familiar with.
Employees	The store treats their employees well.
Eco	The store is environmentally conscious.
Discount	I can get a discount by using a store card.
Delivery	The store delivers to my home.
Coupons	The location accepts coupons.
Clean	I think the store is clean.
Cashiers	The store generally has enough cashiers.
Cart	I can always get a shopping cart.
Bulk	I can buy fruits and vegetables in bulk.
Bag	The store generally has someone to bag my groceries.
Aisle	The aisles of the store are wide enough for me to shop comfortably.
Ads	The store sends out a sale paper or advertisement.

Items were rated on 5-point Likert scales (1 = strongly disagree, 5 = strongly agree) describing attributes of fruit- and vegetable-purchasing locations.

All responses were deidentified and linked to unique participant identifiers. A total of 491 panel respondents were screened for eligibility, of whom 343 were eligible. Of these, 148 caregivers met all inclusion criteria and provided complete data on key analytic variables and were included in the analytic sample. The remaining eligible respondents were excluded due to missing information on >1 key variable. Examination of missingness patterns indicated that excluded observations often reflected partially completed surveys rather than isolated item nonresponse. Missingness was minimal for key continuous variables, moderate for most store-preference items, and higher for several demographic variables (Supplemental Table 1). These data form the basis for the descriptive and regression analyses presented in this paper.

### Exploratory factor analysis

Factor analysis is a statistical method used to identify underlying relationships among observed variables by reducing data dimensionality while retaining key information [16,17]. The method is based on the concept of latent variables—unobserved constructs inferred from correlations among observed measures [18]. It assumes that observed variables share common variance attributable to >1 latent factor, with unique variance reflecting measurement error or variable-specific influences [19].

Two primary forms of factor analysis are common in research: exploratory factor analysis (EFA) and confirmatory factor analysis (CFA). EFA is appropriate for identifying latent structures without imposing a predetermined model, whereas CFA tests hypothesized structures [18]. In this study, EFA was employed to uncover latent dimensions of caregiver shopping preferences without prior assumptions.

EFA can be expressed as

(1) $$\begin{array}{c} x_{i}=\Lambda f_{i}+\epsilon_{i} \end{array}$$

where $x_{i}$ is a vector of the *p* observed store-preference items for respondent *i*, Λ is the *p* × *m* matrix of factor loadings linking each observed variable to the *m* latent factors, $f_{i}$ is a vector of latent factor scores for respondent *i*, and $\epsilon_{i}$ is a vector of unique variances (measurement error and variable-specific effects). The factors $f_{i}$ are assumed to capture the shared variance among the observed items, whereas the unique terms $\epsilon_{i}$ are uncorrelated across items. In this study, the estimated factor scores were standardized and subsequently used as dependent variables in the regression analysis.

The dataset initially contained 38 shopping preference items. One variable — “close to public transportation”—was excluded because >20% of responses were missing, leaving 37 items for analysis. Factor extraction was conducted using the principal factor method with oblique (oblimin) rotation, which allows for correlations among factors [20]. Factor scores were computed using the regression method to generate standardized values for each factor.

The number of retained factors was determined using 3 established criteria. Parallel analysis compared eigenvalues from the observed data to those from randomly simulated datasets, retaining factors whose actual eigenvalues exceeded the simulated values [[20], [21], [22], [23]]. The scree plot provided a visual inspection of eigenvalues in descending order, with factors retained above the “elbow” point that accounted for meaningful variance [19,24,25]. The Kaiser criterion was also applied, retaining factors with eigenvalues >1 [26,27]. These 3 methods were used in combination to reduce the likelihood of overextraction.

### Regression analysis

The EFA retains 5 dimensions of store preference, namely store quality and service, in-store accessibility, online and delivery options, price sensitivity, and food quality and health; these are reported in the factor structure of store preferences section and displayed in Figure 1, and each serves as the dependent variable in one of the models described here. To examine how household characteristics influence each dimension of grocery-store preferences, 5 ordinary least squares (OLS) regression models were estimated, with each of the 5 standardized factor scores from the EFA serving as a dependent variable. This approach quantifies how demographic, socioeconomic, and behavioral characteristics are associated with variation in each latent preference dimension. Formally, the model can be expressed as

(2) $$\begin{array}{c} f_{ki}=\beta_{0k}+X_{i}^{\prime }\beta_{k}+\varepsilon_{ki},k=1,\ldots ,K\text{,} \end{array}$$

where$f_{ki}$ denotes the standardized factor score for preference dimension *k* for household *i*, $X_{i}^{\prime }$ is the vector of household-level predictors, $\beta_{k}$ is the vector of estimated coefficients, and $\varepsilon_{ki}$ is the idiosyncratic error term. Because each equation includes the same covariates, the coefficients $\beta_{k}$ can be interpreted as how the same set of household characteristics relates to distinct aspects of store preferences.

**FIGURE 1:**
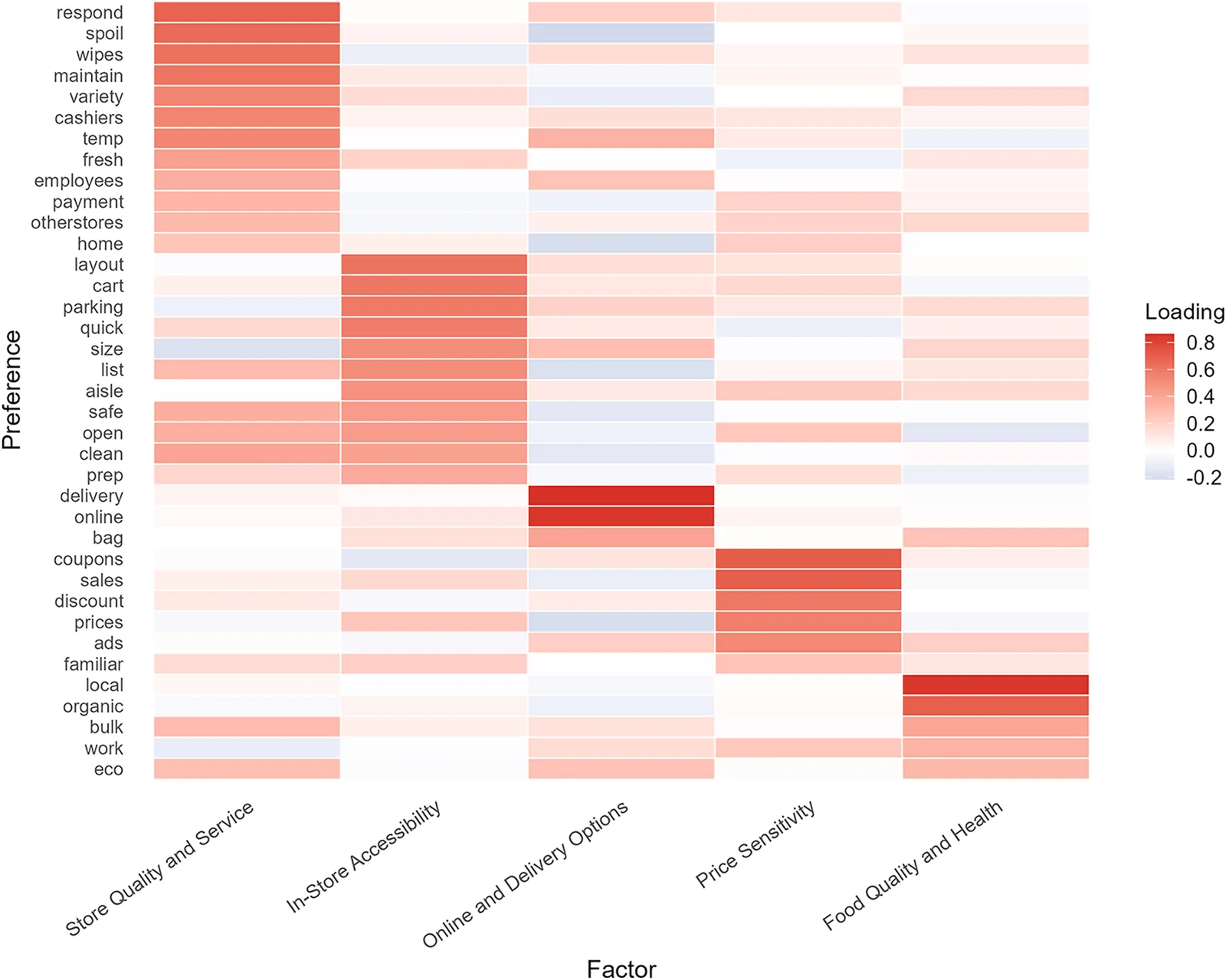
Factor loadings for store-preference items. The heat map presents standardized factor loadings from an exploratory factor analysis of 37 store-preference items. Each row corresponds to a specific store attribute, and each column to 1 latent factor. Darker red shading indicates stronger positive loadings, darker blue shading indicates stronger negative loadings, and white represents loadings near zero. Factors are labeled according to the dominant themes among items with the highest loadings.

The dependent variables were standardized factor scores derived from EFA, so their scales are unitless and relative to the sample. The coefficients describe the direction and relative strength of association between predictors and each preference dimension. All models were estimated using heteroskedasticity-robust SE to account for potential nonconstant variance in the residuals. Although the same predictors were included in each regression, separate estimation allows for factor-specific relationships to emerge across the 5 preference dimensions. The model assumptions were checked as follows. Constant error variance was assessed with a Studentized Breusch–Pagan test, with the variance equation specified in the full covariate set. Influential observations were assessed with Cook’s distance, against a conventional threshold of one. Residual normality was examined. Collinearity was assessed with variance inflation factors against a conventional threshold of 10. The model diagnostics section reports what each of these checks returned.

The vector of covariates $X_{i}^{\prime }$ includes 18 predictors capturing demographic, socioeconomic, and behavioral characteristics. It comprises the sociodemographic characteristics of the developers of the store-selection instrument named as the open question, together with the household food role and program-participation measures the study was designed around, and the same vector enters all 5 equations within a sample, so that the 5 dimensions are compared on identical conditioning. Demographic measures comprise caregiver age in years, number of children in the household, caregiver marital status (single compared with married or partnered), and toddler’s gender. Caregiver ethnicity and race are represented as 4 mutually exclusive categories—Hispanic, non-Hispanic White, non-Hispanic Black, and non-Hispanic Other (combining Asian and Other because of small cell sizes; Table 2)—with non-Hispanic White serving as the reference group; ethnicity and race were collected for the caregiver only, and no race or ethnicity variable was collected for the toddler.

**TABLE 2:** Categorical characteristics, full sample, and mothers-only subsample

Characteristic	Group	Full sample (*n* = 148)		Mothers-only (*n* = 121)	
		Count	%	Count	%
Toddler gender	Girl	80	54.05	67	55.37
	Boy	68	45.95	54	44.63
Caregiver race and ethnicity	Hispanic	45	30.41	43	35.54
	Non-Hispanic White	49	33.11	32	26.45
	Non-Hispanic Black	44	29.73	37	30.58
	Non-Hispanic other	10	6.76	9	7.44
Caregiver marital status	Married/partnered	94	63.51	72	59.50
	Single	54	36.49	49	40.50
Caregiver education level	Less than high school	6	4.05	5	4.13
	High school	114	77.03	97	80.17
	College and higher	28	18.92	19	15.70
Daycare frequency	Not regularly	86	58.11	73	60.33
	Part-time	37	25.00	26	21.49
	Full time	25	16.89	22	18.18
Food shopping frequency	Never	0	0.00	0	0.00
	Occasionally	12	8.11	9	7.44
	Sometimes	3	2.03	2	1.65
	Often	133	89.86	110	90.91
Food preparation frequency	Never	0	0.00	0	0.00
	Occasionally	12	8.11	8	6.61
	Sometimes	8	5.41	5	4.13
	Often	128	86.49	108	89.26
Relationship to toddler	Mother	123	83.11	121	100.00
	Father	16	10.81	—	—
	Other relative	9	6.08	—	—
Caregiver employment status	Yes	—	—	71	58.68
	No	—	—	50	41.32

Caregiver race and ethnicity are reported in the 4 mutually exclusive categories used in the regression models, which combine reported race with Hispanic ethnicity, with non-Hispanic Asian and non-Hispanic Other respondents combined into a single non-Hispanic Other category because of small cell sizes; non-Hispanic White is the reference category. The reported race and Hispanic status distributions from which the 4 categories are formed are given in Supplemental Table 6. Percentages may not total 100% because of rounding. The mothers-only subsample excludes 2 cases with missing employment data.

Caregiver education is represented by binary indicators for high school diploma and college degree or higher, with less than high school as the reference category. Behavioral and program-participation variables include frequency of food shopping, frequency of food preparation, frequency of daycare attendance, and dinner-out frequency, measured as the number of times per week the caregiver eats dinner out with the family. Caregiver age, number of children, daycare frequency, and dinner-out frequency each enters linearly.

For food shopping and preparation, original ordinal responses (“occasionally,” “sometimes,” “often”) were recoded into binary indicators distinguishing “often” from “not often,” based on preliminary analysis showing nonlinear associations with preferences. The 2 indicators overlap substantially: 125 of the 148 caregivers report performing both roles often, agreement between the 2 measures is 92.6%, 11 caregivers are discordant, the phi coefficient is 0.65, and the Spearman correlation across the original 3 response levels is 0.68; among the 121 mothers, the corresponding figures are 105, 93.4%, 8, 0.63, and 0.66. Supplemental Table 2 gives the full cross-classification for both samples. Relationship to the toddler enters the full-sample model as 2 indicators, for father and for other relative, with mother as the reference category. The full-sample models therefore estimate 18 coefficients, every one of which is reported in Supplemental Table 3.

Participation in food assistance programs was measured with binary indicators of SNAP, WIC, and free or reduced-price school meals. These items are asked at the household level and carry a 12-mo reference period, so an affirmative response indicates that the respondent or another member of the household received the benefit in the previous 12 mo. Participation in free or reduced-price school meals therefore indicates a school-age member of the household rather than participation by the toddler, and, for a respondent surveyed between March and July 2022, the 12-mo window covers the 2021 to 2022 school year in full. Because participation in ≥1 of 8 federal food assistance programs was an eligibility criterion and the models carry indicators for 3 of them, each of these indicators contrasts caregivers who receive that benefit with caregivers who receive ≥1 other form of federal food assistance rather than with caregivers who receive none: of the 37 caregivers who do not report SNAP, every one reports ≥1 of the other 7 programs. Employment status was excluded from the full-sample model because of ambiguity in the survey question, specifically whether a “yes” response indicated one or both parents were employed in dual-parent households.

Because mothers represented >80% of the sample and are often the primary decision makers in household food purchasing, a separate regression analysis was conducted for this subgroup. This model retains every predictor of the full-sample model except the 2 relationship-to-toddler indicators, which are constant within the subsample, and adds a binary employment status variable to assess whether working mothers differ from nonworking mothers in their store preferences. The mothers-only models therefore estimate 17 coefficients, all of which are reported in Supplemental Table 4. This addition is particularly relevant for preference dimensions such as online and delivery options and price sensitivity, which may be more strongly influenced by time constraints and budgeting considerations among working mothers. The same mutually exclusive ethnicity and race categories used in the full-sample analysis were applied to the mothers-only subgroup. This analysis allowed us to determine whether the relationships observed in the overall sample hold for mothers specifically and to assess whether employment moderates store preferences in ways that could inform targeted interventions. The distribution of ethnicity and race in the mothers-only sample is also provided in Table 2.

### Sample size considerations

The analytic sample comprised 148 caregivers. The study was designed to characterize patterns in caregivers’ evaluations of FV purchasing locations and to examine how these patterns relate to household characteristics. Accordingly, EFA was used to summarize a broad set of store attributes into a smaller number of latent dimensions, followed by regression analyses relating standardized factor scores to caregiver and household characteristics. These analyses are intended to describe and organize preference structures in this population rather than to test prespecified causal hypotheses.

Because several of the contrasts the analysis draws on small cells, we report the approximate effect size each contrast can detect. The calculation sets the 2-sided significance level at 5% and power at 80%, and it takes the SE of each contrast from the fitted multivariable model for each dimension, so that the residual variance and the conditioning set are those of the estimated models rather than those of a 2-group comparison. The resulting approximate minimum detectable difference is expressed in standard deviations of the dimension, and the 5 per-dimension figures are averaged with equal weight. On that basis, the full sample detects a difference of ∼0.37 SDs of the outcome between caregivers of girls and caregivers of boys, 0.39 for WIC participation, 0.41 for participation in free or reduced-price school meals, 0.42 for single against married or partnered caregivers, 0.45 for SNAP participation, 0.49 for Hispanic against non-Hispanic White caregivers, and 0.50 for non-Hispanic Black against non-Hispanic White caregivers. It requires ∼0.80 SDs for the non-Hispanic Other category, which contains 10 caregivers, and 0.81 for the other relative category, which contains 9, and ∼1.05 SDs for the contrast between caregivers with a college degree and caregivers with less than a high school education, whose reference cell contains 6. The 2 household food-role contrasts require ∼0.74 SDs for food preparation and 0.84 for food shopping in the full sample, and 0.86 and 0.95 among mothers. Among mothers, the corresponding figures for the demographic contrasts are larger throughout, reaching 1.19 SDs for the education contrast. Every coefficient is reported with its 95% confidence interval (CI): Figures 2 and 3 draw 90% and 95% intervals around every estimated association in the full-sample and mothers-only models, and Supplemental Tables 3 and 4 report the estimate, the SE, the 95% CI, and the *P* value for every term in every equation. Given the exploratory nature of these analyses, it should be interpreted descriptively alongside the effect estimates and CIs.

**FIGURE 2:**
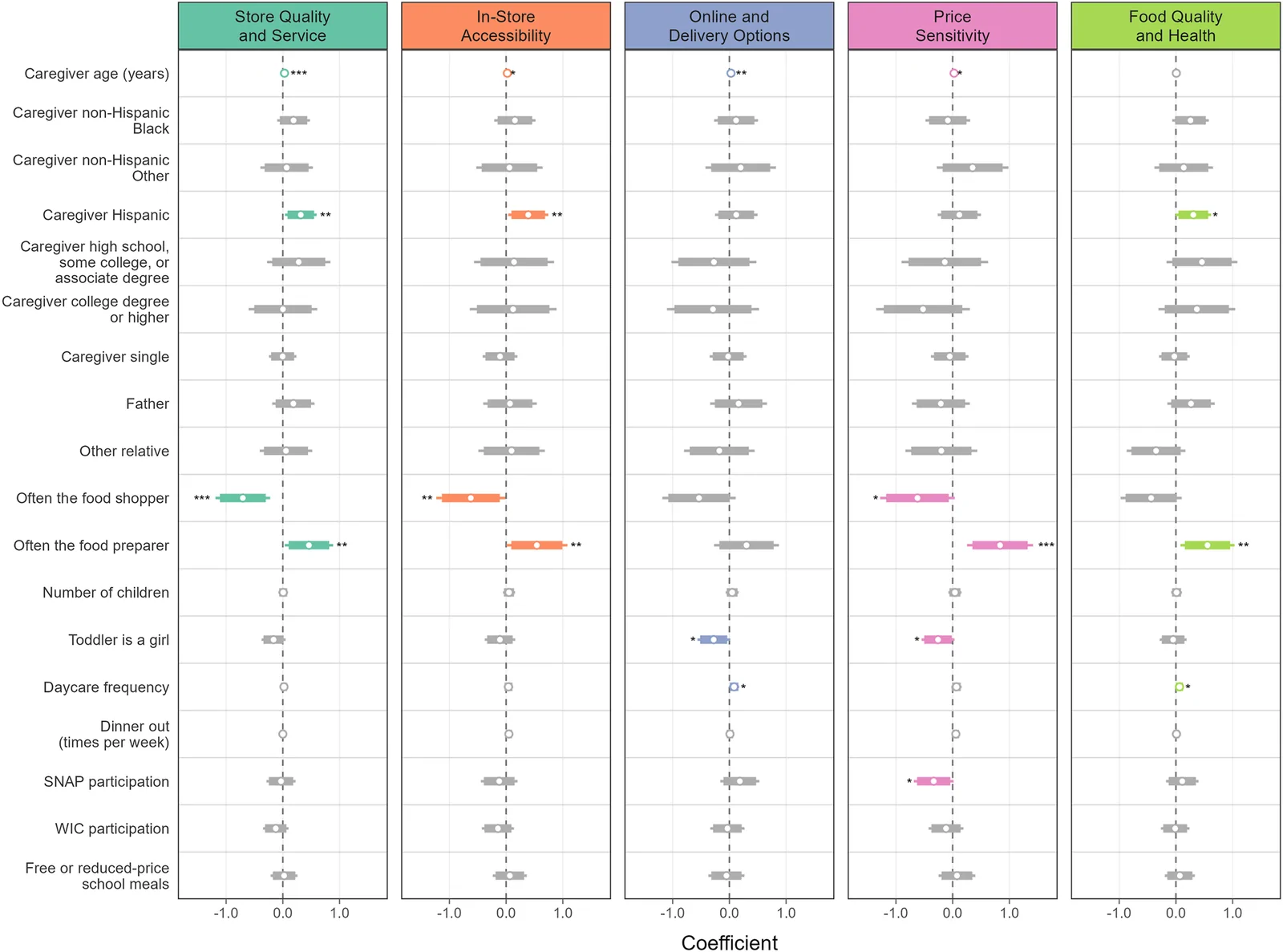
Estimated coefficients of caregiver, child, and household characteristics on factor scores, full sample. Coefficients are estimates from separate ordinary least squares regression models for each factor using the full sample (*n* = 148). The figure is arranged in 5 panels, one for each store-preference dimension, and every covariate included in the model appears in every panel, in the same order, so that all 90 estimated associations are displayed. Positive values indicate higher factor scores associated with the predictor, whereas negative values indicate lower scores. Caregiver age, number of children, daycare frequency, and dinner out are continuous covariates drawn per 1-unit change, and their estimates are therefore not comparable in magnitude to the binary contrasts drawn on the same axis. For each association, the marker is the point estimate, the thick band is the 90% CI, and the thin band is the 95% CI, both centered on the point estimate. An association with *P* ≥ 0.10 is drawn in gray, and an association with *P* < 0.10 is drawn in the color of its dimension. The dashed vertical line marks zero. The numerical values of all coefficients, with SEs, 95% CIs, and *P* values, are reported in Supplemental Table 3. Stars denote ∗*P* < 0.10, ∗∗*P* < 0.05, and ∗∗∗*P* < 0.01. Reference categories for the contrasts shown are caregiver non-Hispanic White, caregiver less than high school, caregiver married or partnered, caregiver is the mother, and toddler is a boy. CI, confidence interval; SNAP, Supplemental Nutrition Assistance Program; WIC, Special Supplemental Nutrition Program for Women, Infants, and Children.

**FIGURE 3:**
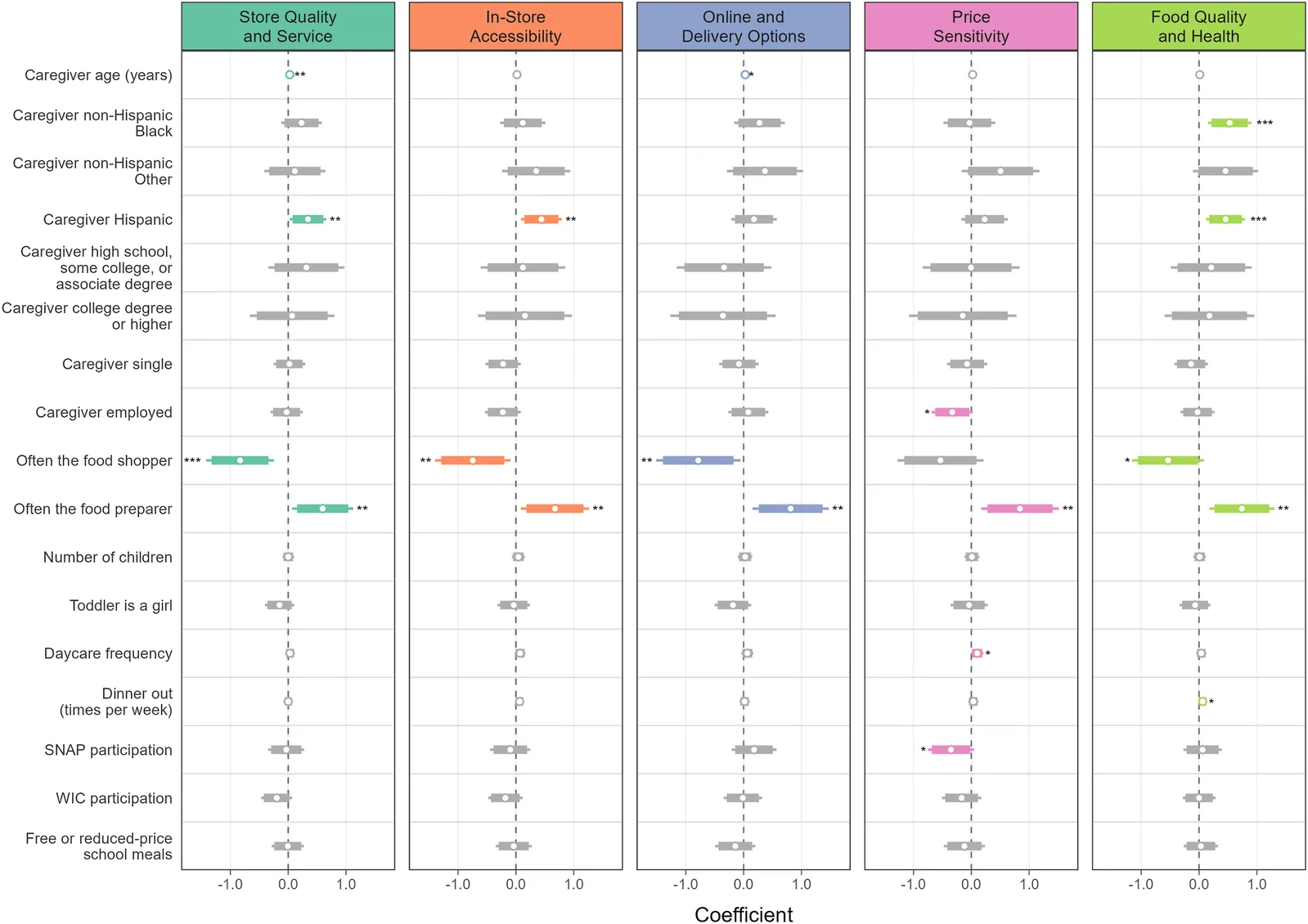
Estimated coefficients of caregiver, child, and household characteristics on factor scores, mother-only. Coefficients are estimates from separate ordinary least squares regression models for each factor using the mothers-only subsample (*n* = 121). The figure is arranged in 5 panels, one for each store-preference dimension, and every covariate included in the model appears in every panel, in the same order, so that all 85 estimated associations are displayed. Positive values indicate higher factor scores associated with the predictor, whereas negative values indicate lower scores. Caregiver age, number of children, daycare frequency, and dinner out are continuous covariates drawn per 1-unit change, and their estimates are therefore not comparable in magnitude to the binary contrasts drawn on the same axis. For each association, the marker is the point estimate, the thick band is the 90% CI, and the thin band is the 95% CI, both centered on the point estimate. An association with *P* ≥ 0.10 is drawn in gray, and an association with *P* < 0.10 is drawn in the color of its dimension. The dashed vertical line marks zero. The numerical values of all coefficients, with SEs, 95% CIs, and *P* values, are reported in Supplemental Table 4. Stars denote ∗*P* < 0.10, ∗∗*P* < 0.05, and ∗∗∗*P* < 0.01. Reference categories for the contrasts shown are caregiver non-Hispanic White, caregiver less than high school, caregiver married or partnered, caregiver not employed, and toddler is a boy. CI, confidence interval; SNAP, Supplemental Nutrition Assistance Program; WIC, Special Supplemental Nutrition Program for Women, Infants, and Children.

## Results

### Factor structure of store preferences

EFA yielded a 5-factor solution that achieved an optimal balance between statistical fit and interpretability. Although the heterogeneous set of 37 store-attribute items produced modest sampling adequacy, Bartlett’s test of sphericity indicated that the correlation matrix was suitable for factor analysis (*χ*^2^[666] = 13,095.3; *P* < 0.001). Five factors were retained based on parallel analysis and interpretability, explaining 57% of the total variance. Internal consistency for the 5 latent dimensions was acceptable to excellent, with Cronbach’s *α* ranging from 0.72 to 0.88 and McDonald’s $\omega_{h}$ ranging from 0.74 to 0.89.

The factor loading matrix, visualized in Figure 1, depicts the magnitude and direction of association between each store-preference item and the extracted latent factors. The heatmap uses a red–white–blue color gradient, with darker red indicating strong positive loadings, white indicating near-zero loadings, and blue indicating negative loadings. This visual format enables rapid identification of the items most strongly associated with each factor, as well as any cross-loading patterns.

The first factor, store quality and service, is characterized by high loadings on items related to store cleanliness, maintenance, employee helpfulness and responsiveness, and perceived safety. This factor reflects the importance caregivers place on well-maintained and professionally managed stores that provide reliable service during food shopping trips.

The second factor, in-store accessibility, loads strongly on items related to ease of navigation and physical comfort within the store, including familiarity with store layout, availability of shopping carts and parking, aisle width, store size, and the ability to locate FVs efficiently. These loadings capture caregivers’ preferences for shopping environments that reduce physical and cognitive effort while shopping.

The third factor, online and delivery options, is defined by high loadings on the availability of online ordering and home delivery. This factor reflects caregivers’ preferences for digital shopping modes that allow groceries to be obtained without an in-store visit.

The fourth factor, price sensitivity, is characterized by strong loadings on items related to discounts, competitive pricing, and value for money. It reflects shoppers’ attention to cost considerations and promotional price advantages.

The fifth factor, food quality and health, is distinguished by high loadings on preferences for organic produce, freshness, local sourcing, and environmentally sustainable practices. This factor captures a values-oriented dimension of store choice grounded in health, nutrition, and environmental responsibility.

Collectively, these 5 latent factors capture the multifaceted nature of caregiver shopping preferences. They explain the underlying covariance structure among the 37 preference items and serve as dependent variables in subsequent regression analyses. The heatmap in Figure 1 thus provides both a diagnostic check on the factor structure and an intuitive summary of how specific store attributes align with each underlying dimension.

### Descriptive statistics: full sample

The analytic sample comprised 148 caregivers of toddlers in the Dallas–Fort Worth area, and summary statistics are presented in the full-sample columns of Tables 2 and 3, and Supplemental Table 5. Most respondents were mothers (83%), followed by fathers (11%), and other relatives such as grandparents, aunts, or uncles (6%). Slightly more than half of the toddlers were girls (54%). Caregiver ethnicity and race are reported once, in the 4 mutually exclusive categories used in the regression models: 30% of caregivers were Hispanic, 33% non-Hispanic White, 30% non-Hispanic Black, and 7% non-Hispanic Other. Non-Hispanic White is the reference category in all models, and Supplemental Table 6 gives the reported race and Hispanic status responses from which the 4 categories are formed.

**TABLE 3:** Continuous caregiver, child, and household characteristics, full sample, and mothers-only subsample

Variable	Full sample (*n* = 148)					Mothers-only (*n* = 121)				
	Mean	Min	Median	Max	SD	Mean	Min	Median	Max	SD
Caregiver age (y)	30.68	18.00	30.00	54.00	6.74	30.12	18.00	30.00	47.00	6.17
Toddler age (mo)	26.03	14.00	27.00	40.00	10.41	25.39	14.00	27.00	40.00	10.29
Number of children	2.44	1.00	2.00	7.00	1.41	2.41	1.00	2.00	7.00	1.36
Dinner out (times/wk)	3.20	1.00	3.00	17.00	2.10	3.08	1.00	2.00	11.00	1.79

The mothers-only subsample excludes 2 cases with missing employment data.

Regarding household structure, 64% of caregivers were married or partnered, and 36% were single. Educational attainment was relatively high: 77% had completed high school as their highest level of education, and 19% held a college degree or higher. Caregiver engagement in food-related tasks was substantial, with 90% shopping for food often and 86% preparing meals often. Daycare arrangements varied: 58% of toddlers were not regularly enrolled in daycare, 25% attended part-time, and 17% attended full-time.

The mean caregiver age was 30.68 y (SD = 6.74), and the mean toddler age was 26.0 mo (median 27, range, 14–40, SD = 10.4), which sits inside the eligibility window of 12 to 47 mo. There was a mean of 2.44 children (SD = 1.41) per household and caregivers reported eating dinner out 3.20 times per week (SD = 2.10). Participation in ≥1 federal food assistance program was an eligibility criterion, so every caregiver in the sample participated by design, and the informative quantity is the composition of participation conditional on that screen. Conditional on the screen, 75.0% of caregivers reported SNAP, 39.2% reported WIC, and 31.8% reported free or reduced-price school meals; 52.7% reported 1 program, 31.8% 2, 9.5% 3, and 6.1% ≥4.

Bivariate comparisons using *F* tests identified several noteworthy subgroup differences, which are reported in full in Supplemental Table 7. Caregivers of boys had slightly higher online and delivery options scores than caregivers of girls (*P* < 0.10). Hispanic respondents scored higher on store quality and service (*P* < 0.10) and on in-store accessibility (*P* < 0.05) than non-Hispanic respondents. Although racial differences were generally small, caregivers identifying as Asian or Other reported descriptively higher price sensitivity scores. Marital status and education level were not significantly associated with preference scores, although caregivers with only a high school education reported slightly higher food quality and health and price sensitivity scores. Frequency of food preparation was associated with store preferences: caregivers who prepared meals often had higher price sensitivity scores (*P* < 0.05), and in-store accessibility scores differed across preparation frequency groups (*P* < 0.10).

### Descriptive statistics: mothers-only subsample

The mothers-only subsample included 121 respondents (82% of the full sample) after excluding 2 mothers with missing employment data; summary statistics are presented in the mothers-only columns of Tables 2 and 3, and Supplemental Table 5.

Overall, the demographic and household characteristics of mothers closely mirrored those of the full sample. Most mothers were married or partnered, had completed at least high school, and reported frequent engagement in food shopping and preparation. Daycare use and the distribution of toddler gender were also similar to the full-sample patterns. Approximately 59% of mothers were employed.

Continuous characteristics likewise aligned with the full sample: mothers averaged 30.12 y of age (SD = 6.17), households had 2.41 children on average (SD = 1.36), and mothers reported eating dinner out 3.08 times per week (SD = 1.79).

Participation was likewise a condition of the design in this subsample, and its composition closely reflects the full sample: 75.2% of mothers reported SNAP, 38.8% reported WIC, and 31.4% reported free or reduced-price school meals.

### Regression results: full sample

In the full-sample OLS regression analysis, several caregiver and household characteristics were associated with the 5 latent store-preference dimensions at *P* < 0.10 (Figure 2; full coefficient estimates in Supplemental Table 3). Caregiver age was positively associated with store quality and service (*β* = 0.03, *P* < 0.01), in-store accessibility (*β* = 0.02, *P* < 0.10), online and delivery options (*β* = 0.03, *P* < 0.05), and price sensitivity (*β* = 0.03, *P* < 0.10). Caregivers of girls reported lower scores for online and delivery options (*β* = −0.28, *P* < 0.10) and price sensitivity (*β* = −0.26, *P* < 0.10) than caregivers of boys.

Hispanic caregivers had higher preferences for store quality and service (*β* = 0.32, *P* < 0.05), in-store accessibility (*β* = 0.39, *P* < 0.05), and food quality and health (*β* = 0.31, *P* < 0.10). Frequent food preparation was positively associated with store quality and service (*β* = 0.46, *P* < 0.05), in-store accessibility (*β* = 0.54, *P* < 0.05), price sensitivity (*β* = 0.84, *P* < 0.01), and food quality and health (*β* = 0.56, *P* < 0.05). Frequent food shopping was negatively associated with store quality and service (*β* = −0.71, *P* < 0.01), in-store accessibility (*β* = −0.62, *P* < 0.05), and price sensitivity (*β* = −0.62, *P* < 0.10). SNAP participation was negatively associated with price sensitivity (*β* = −0.33, *P* < 0.10). Greater daycare attendance was associated with higher scores for online and delivery options (*β* = 0.08; 95% CI: −0.01, 0.17; *P* < 0.10) and food quality and health (*β* = 0.06; 95% CI: −0.01, 0.14; *P* < 0.10).

Because the dependent variables are standardized, each coefficient is a difference in standard deviations of its dimension, and on that scale, these associations range from small to large. The association with caregiver age is small: across the IQR, from 26 to 34 y, the implied difference is 0.23 SDs of store quality and service. The demographic and behavioral contrasts are larger. Hispanic caregivers score 0.32 higher on store quality and service than non-Hispanic White caregivers and 0.39 higher on in-store accessibility, and SNAP participation corresponds to 0.33 less price sensitivity. The 2 household food-role contrasts are the largest in the model, at 0.84 more price sensitivity for frequent food preparation and 0.71 less store quality and service for frequent food shopping. Figure 2 draws each association with its CI, and Supplemental Table 3 reports every coefficient with its 95% CI and its *P* value.

WIC participation was included in every model, and we did not reject the null of no association with any dimension at *P* < 0.10 in the full sample. The estimates are small and imprecisely determined: −0.12 on store quality and service (95% CI: −0.35, 0.10), −0.14 on in-store accessibility (95% CI: −0.43, 0.14), −0.03 on online and delivery options (95% CI: −0.34, 0.27), −0.12 on price sensitivity (95% CI: −0.42, 0.19), and −0.01 on food quality and health (95% CI: −0.27, 0.24). These intervals are wide enough to be consistent with associations of either sign, so they indicate imprecision rather than the absence of a relationship. Because food shopping and food preparation overlap for most caregivers, we also reestimated all 10 equations with the 2 indicators replaced by a single combined variable, in each of the 3 forms such a combination can take: an indicator for caregivers who perform both roles often, a count of how many of the 2 roles the caregiver performs often, and the net contrast between caregivers who perform both roles and caregivers who perform neither. None of the 3 produces a role coefficient significant at *P* < 0.10 in any of the 10 equations, and Supplemental Table 8 reports all of them. Joint tests on the retained 2-variable specification account for that pattern: the null that both role coefficients are zero is rejected in 9 of the 10 equations at the 10% level, the restriction that the 2 coefficients are equal is rejected in 9 of the 10 at the 5% level, and the restriction that they are equal in magnitude and opposite in sign is not rejected in any equation. None of the 3 combined variables separates the 2 roles: the count and the net contrast each estimate the sum of the 2 coefficients, and the both-roles indicator marks the caregivers who carry both. The contrast between the 2 roles is identified from the 11 caregivers who perform one role often and not the other and is estimated with correspondingly little precision.

### Regression results: mothers-only subsample

In the mothers-only models (Figure 3; full coefficient estimates in Supplemental Table 4), caregiver age was positively associated with store quality and service (*β* = 0.03, *P* < 0.05) and online and delivery options (*β* = 0.03, *P* < 0.10). Hispanic mothers had higher scores for store quality and service (*β* = 0.34, *P* < 0.05), in-store accessibility (*β* = 0.44, *P* < 0.05), and food quality and health (*β* = 0.46, *P* < 0.01). Non-Hispanic Black mothers had higher food quality and health scores (*β* = 0.53, *P* < 0.01).

Frequent food preparation was positively associated with all 5 preference factors: store quality and service (*β* = 0.60, *P* < 0.01), in-store accessibility (*β* = 0.68, *P* < 0.01), online and delivery options (*β* = 0.81, *P* < 0.05), price sensitivity (*β* = 0.84, *P* < 0.05), and food quality and health (*β* = 0.74, *P* < 0.01). Frequent food shopping was negatively associated with store quality and service (*β* = −0.83, *P* < 0.01), in-store accessibility (*β* = −0.75, *P* < 0.05), online and delivery options (*β* = −0.79, *P* < 0.05), and food quality and health (*β* = −0.54, *P* < 0.10). Daycare attendance frequency was positively associated with price sensitivity (*β* = 0.10, *P* < 0.10), and dinner-out frequency was positively associated with food quality and health (*β* = 0.06, *P* < 0.10). Employment was negatively associated with price sensitivity (*β* = −0.33, *P* < 0.10), as was SNAP participation (*β* = −0.35, *P* < 0.10).

The pattern among mothers matches the full sample, and the magnitudes are somewhat larger. Non-Hispanic Black mothers score 0.53 SD higher on food quality and health than non-Hispanic White mothers, and Hispanic mothers 0.44 SD higher on in-store accessibility. Frequent food preparation corresponds to 0.84 more price sensitivity, and frequent food shopping to 0.83 less store quality and service. Across the IQR of maternal age, from 26 to 33 y, the implied difference is 0.19 SDs of store quality and service. FIGURE 2, FIGURE 3 draw each association with its 90% and 95% CIs, and Supplemental Table 4 reports every coefficient in the 5 models. WIC participation was likewise included in every mothers-only model, and we did not reject the null of no association with any dimension at *P* < 0.10. The estimates are −0.20 on store quality and service (95% CI: −0.47, 0.07), −0.18 on in-store accessibility (95% CI: −0.48, 0.11), −0.01 on online and delivery options (95% CI: −0.35, 0.32), −0.17 on price sensitivity (95% CI: −0.51, 0.17), and 0.00 on food quality and health (95% CI: −0.28, 0.29). As in the full sample, these intervals reflect imprecision rather than the absence of a relationship.

### Model diagnostics

The Breusch–Pagan test marginally rejects constant error variance in 1 of the 10 equations, price sensitivity in the mothers-only subsample (*P* = 0.075), and does not reject in the remaining 9, where *P* ranges from 0.122 to 0.676; no equation rejects at the 5% level. No observation exceeds a Cook’s distance of one in any equation, the largest value in either sample being 0.18. Residuals depart from normality in most equations. The largest variance inflation factor in either sample is 5.38, and for the 2 household food-role indicators, the factor is 2.03 for food shopping and 2.04 for food preparation in the full sample, and 2.02 and 1.91, respectively, among mothers.

## Discussion

This study describes how low-income caregivers of toddlers who participate in federal food assistance weigh the attributes of the stores where they purchase FVs. Our analysis considers a more comprehensive store-attribute battery rather than focusing solely on price and proximity. The 5 dimensions recovered here show the diverse and sometimes contrasting ways in which caregivers of toddlers prioritize store attributes when making food purchasing decisions. Rather than revealing a uniform pattern, the findings suggest that store preferences are shaped by household roles, sociodemographic characteristics, and everyday constraints. Several associations reported are consistent with patterns already established in the literature on household food purchasing, and they provide evidence that the dimensions behave as expected in a population in which this instrument had not previously been applied.

Our study distinguishes between food preparation and food shopping responsibilities. Across both the full sample and the mothers-only subgroup, frequent food preparers consistently expressed stronger preferences for store quality and service, in-store accessibility, price sensitivity, and food quality and health; among mothers, this pattern extended to all 5 preference dimensions, including online and delivery options. These results align with prior research showing that hands-on cooking promotes more deliberate and health-conscious food decisions [28]. Similarly, frequent food shoppers displayed weaker preferences across nearly all dimensions, reflecting a more routine-driven, efficiency-oriented mindset [29]. Such routinized shopping behavior may also reduce attention to store features such as service quality, shopping ease, or pricing, a pattern documented in retail studies of habit-driven grocery trips [[30], [31], [32]]. The 2 role indicators have different coefficients in the mutually adjusted models, although the contrast between them rests on the 11 discordant caregivers and is estimated with limited precision. The 2 role coefficients are also statistically indistinguishable from being equal and opposite, which helps explain why none of the combined variables reported are statistically distinguishable from zero.

Parental age was a consistent positive predictor of store quality and service and online and delivery options preferences. In the full sample, older caregivers also valued in-store accessibility and price savings, whereas among mothers, the association was more limited. Because little existing research compares how maternal and nonmaternal caregivers evaluate store attributes, this age-related pattern may represent an area for future investigation into role-specific shopping behaviors.

Time constraints showed different implications across models. In the full sample, greater daycare use was associated with higher preferences for online and delivery options and food quality and health, consistent with evidence that time scarcity can promote streamlined yet health-conscious purchasing [33,34]. Among mothers, however, daycare attendance predicted greater price sensitivity, suggesting that structured childcare schedules may heighten budget considerations for primary food decision makers.

Dinner-out frequency also differentiated preferences. Among mothers, eating dinner out more often was associated with higher priorities for food quality and health, a relationship that was not observed in the full sample. One possible explanation is that dinner-out frequency may proxy for greater household resources or discretionary income, as higher-income households tend to eat out more frequently, which may reduce budget constraints and allow caregivers to place greater emphasis on food quality and health when shopping [35,36]. Alternatively, greater exposure to a wider range of food environments through eating dinner out may shape expectations for freshness and quality, influencing preferences for higher-quality food options [28,29].

Toddler gender predicted shopping preferences in the full sample: caregivers of girls expressed lower preferences for online and delivery options and price sensitivity. The corresponding associations among mothers are smaller and are not statistically distinguishable from zero, and the mothers-only estimates are reported with their CIs in Supplemental Table 4. Prior work has shown that boys are more often placed in lower-quality childcare settings [37], potentially increasing time pressures that influence shopping behavior.

Ethnic and racial identity also shaped store preferences in distinctive ways. Hispanic caregivers consistently placed greater importance on store quality and service, in-store accessibility, and food quality and health. This pattern aligns with research showing that low-income Hispanic families often maintain stronger access to and valuation of fresh FVs in the home environment compared with other racial or ethnic groups, such as African American families [38]. Evidence from national samples further indicates that United States Hispanics who shop primarily in supermarkets—where produce quality and availability are highest—have higher FV intake than those relying on convenience outlets [39].

The associations between Hispanic ethnicity and store quality and service and between Hispanic ethnicity and in-store accessibility are also present among mothers. For non-Hispanic Black caregivers, group differences were not evident in the full sample, but Black mothers showed a strong preference for food quality and health. Qualitative research similarly finds that Black and Hispanic parents in underresourced communities emphasize concerns about produce freshness, safety, and the reliability of local food outlets when making purchasing decisions [40].

These racial and ethnic patterns are consistent with broader structural evidence documenting disparities in consumer food environments across minority communities [41], including reduced access to high-quality stores and greater exposure to lower-quality retail options. Importantly, our findings indicate that these disparities do not reflect a lack of demand for nutritious foods; rather, caregivers in these communities express strong preferences for food quality and health, pointing to unmet demand and opportunities to expand access to healthier food options. Moreover, disaggregating analyses by caregiving role reveals meaningful differences that would be obscured in aggregated samples, underscoring the importance of accounting for both cultural context and household responsibilities when evaluating food purchasing behavior.

Economic factors were also influential. SNAP participation was associated with lower price sensitivity in both models. Because participation in ≥1 federal food assistance program was an eligibility criterion, this association compares caregivers who receive SNAP with caregivers whose assistance comes through other programs, most often WIC or free or reduced-price school meals, rather than with caregivers who receive no assistance at all. SNAP supplies a broader and generally larger food budget than those programs do, and the available evidence indicates that SNAP benefits are spent on food rather than substituted toward other consumption, so that participation raises the resources a household has available for food purchases [42,43]. In the mothers-only sample, employment status likewise predicted reduced-price sensitivity. Prior work suggests that lower price sensitivity—whether due to stable benefit timing or wage income—is associated with healthier food purchasing patterns, as households with more flexible food budgets are more likely to buy nutrient-dense items such as FVs and less likely to rely on low-cost, energy-dense alternatives [44]. This relationship is consistent with broader evidence that FV purchases rise with income and that reduced reliance on price promotions is linked to healthier purchasing profiles [45,46].

Overall, these findings emphasize that food purchasing preferences cannot be fully understood through static demographic categories alone. Caregiving behaviors, and in particular the division between preparing and shopping for food, are associated with store priorities, although the contrast between the 2 roles rests on the 11 discordant caregivers and is estimated with limited precision. Subgroup analysis reveals that variables such as toddler gender, daycare use, dining habits, and employment take on different meanings depending on who is making food decisions.

These insights carry several practical implications for both retailers and policymakers. For retailers, the strong and consistent influence of food preparation—especially among mothers—indicates that primary preparers are highly responsive to store quality, in-store accessibility, pricing, and nutrition. This group may be effectively reached through promotions featuring nutrient-dense, affordable items and in-store or online messaging that highlights quick, healthy meal solutions. At the same time, many caregivers in our sample both prepare food and shop frequently, suggesting that these behaviors represent overlapping roles rather than distinct customer types. The negative associations observed for frequent shopping may therefore reflect the context or purpose of specific shopping trips, such as routine, time-pressured visits—rather than stable shopper categories. For retailers, this implies that interventions might need to be tailored to the shopping occasion as much as to the shopper. Simplified store layouts, bulk purchase discounts, and automated shopping tools may be most useful during routine or efficiency-driven trips, whereas quality, branding, and nutrition-oriented messaging may be more effective when caregivers are making planning-oriented or meal-preparation decisions. This perspective aligns with retail research showing that consumers adopt different decision-making strategies depending on shopping occasion (e.g., major stock-up compared with fill-in trips), even within the same household [[30], [31], [32]].

For policymakers and food program planners, the findings reinforce the importance of aligning support with caregivers’ daily routines and shopping priorities. SNAP participation is associated with lower price sensitivity in both models, and food assistance programs more generally stabilize food access, enabling households to maintain regular, nutritious purchases despite market fluctuations. Strengthening these programs to better match caregivers’ schedules and food preparation habits—such as providing targeted incentives for healthy, ready-to-cook items—could increase their effectiveness.

WIC merits particular attention in this population. Every household in the sample contains a child aged 12 to 47 mo and met the study’s food assistance participation screen, and 39% report participating in WIC. The program therefore bears directly on a substantial share of these caregivers, although eligibility was not itself determined in the survey. The survey was fielded between March and July 2022, when the WIC cash-value benefit for FVs stood at $24 per child per month against a pre-pandemic level of $9, so participating caregivers held more FV purchasing power through the program than at any earlier point in its history [47]. Whether that benefit is used depends in part on where it can be spent: evidence from Massachusetts shows that the vendor type a WIC household relies on predicts redemption of the cash-value benefit, with households relying on small grocery stores redeeming at lower rates than those relying on supermarkets [48]. Our results describe what makes a store usable to caregivers in exactly this population, and 2 of the 5 dimensions identified here, store quality and service and in-store accessibility, describe attributes that vendor authorization criteria, which turn on stocking requirements and price, do not address. Whether authorized vendor networks assembled with those attributes in view would raise the share of the benefit that reaches FVs is a question these results suggest is worth evaluating, because the present design observes neither vendor authorization nor redemption.

Childcare dynamics also merit attention. In both the full sample and the mother subgroup, daycare attendance was linked to distinct purchasing preferences, underscoring the influence of time constraints on shopping behavior. Flexible, time-saving food access strategies—such as curbside pickup aligned with childcare drop-off, coordinated meal bundles for busy evenings, or benefits that can be redeemed through streamlined ordering systems—could reduce logistical barriers to healthy food acquisition.

Taken together, these results support moving away from one-size-fits-all approaches toward tailored, context-specific strategies that reflect the diversity of household food decision making. Recognizing the interplay among economic conditions, caregiving roles, and daily routines can help design retail and policy interventions that improve both the accessibility and quality of food available to families with young children.

Several limitations should be considered when interpreting these findings. First, the sample was drawn from the Dallas–Fort Worth metro area, which may limit generalizability to caregivers in other geographic, economic, or cultural contexts. Second, store-preference measures were self-reported and therefore subject to potential social desirability bias, recall error, or hypothetical bias. The study elicits stated preferences over store attributes rather than preferences revealed by observed transactions, so the importance a caregiver reports for an attribute need not equal the weight that attribute carries in an actual store choice. For attributes such as cleanliness, staff conduct, and ease of navigation, however, self-report is the only feasible instrument because these are not recoverable from transaction records. Third, the relatively small sample sizes for both the full sample and the mothers-only subgroup reduce statistical power, making it more difficult to detect smaller effects. Finally, incomplete survey responses further reduced the number of valid observations for some variables, which could introduce bias and affect the precision of estimates. The size of several subgroups further limits what the comparisons can support: only 10 caregivers fall in the non-Hispanic Other category, 9 are other relatives, 6 have less than a high school education, and 11 perform 1 household food role often and not the other. As sample size considerations section reports, the design detects differences of between 0.37 and 0.50 SDs on the well-populated contrasts but requires between 0.80 and 1.05 SDs on these, so a null result on any of them should not be read as evidence of no difference.

In conclusion, despite the aforementioned constraints, this study contributes valuable evidence on the diverse factors influencing caregivers’ food purchasing decisions and underscores the need for more tailored interventions. Future research might consider building on these findings with larger and more diverse samples and incorporating behavioral or longitudinal data to better understand how household dynamics and food environments interact over time. Such efforts could strengthen the evidence base for designing both retail and policy strategies that are equitable, adaptable, and effective in promoting healthy food access for families with young children.

## Author contributions

The authors’ responsibilities were as follows – AL: contributed to conceptualization (equal), formal analysis (lead), methodology (equal), visualization (lead), and writing—original draft preparation (lead); XW: contributed to conceptualization (equal), formal analysis (support), visualization (support), and writing—reviewing and editing (equal); SR: contributed to conceptualization (support) and writing—reviewing and editing (equal); YYZ: contributed to conceptualization (support), supervision (equal), and writing—review and editing (equal); ALMU: contributed to conceptualization (equal), funding acquisition (lead), investigation (lead), methodology (equal), project administration (lead), supervision (equal), visualization (support), and writing—reviewing and editing (equal); and all authors: read and approved the final manuscript.

## Data Availability

Data described in the manuscript, code book, and analytic code will be made available upon request pending application and approval through the corresponding author.

## Declaration of Generative AI and AI-Assisted Technologies in the Writing Process

The author(s) declare that no generative AI or AI-assisted technologies were used in the writing of this manuscript.

## Funding

Funding for this study was provided by NIH NCI Geographic Management of Cancer Health Disparities Program (GMaP).

## Conflict of interest

The authors report no conflicts of interest.
