# Supplementary material for: Multidimensional Grocery-Store Preferences of Low-Income Caregivers of Toddlers
Source: Curr Dev Nutr. 2026 Sep 12;10(10):109530. doi: 10.1016/j.cdnut.2026.109530 (PMC13671030; doi:10.1016/j.cdnut.2026.109530)
Supplement: Supplementary file 1 [file mmc1.docx]

**Supplemental Material**

Article Title:

Multidimensional Grocery-Store Preferences of Low-Income Caregivers of Toddlers

Author Names:

Aihui Li^a^; Xingguo Wang^b^; Stephanie Rogus^c^; Yu Yvette Zhang^d^; Alexandra L. MacMillan Uribe^e^

Affiliations:

^a^ Department of Agricultural Economics, Texas A&M University, 600 John Kimbrough Blvd, College Station, TX, USA. [aihui.li@ag.tamu.edu](mailto:aihui.li@ag.tamu.edu)

^b^ Department of Agricultural Economics, Texas A&M University, 801 Cherry St, Fort Worth, TX, USA. [xingguo.wang@ag.tamu.edu](mailto:xingguo.wang@ag.tamu.edu)

^c^ Texas A&M AgriLife Research at El Paso, 1380 A and M Cir, El Paso, TX, USA. [stephanie.rogus@ag.tamu.edu](mailto:stephanie.rogus@ag.tamu.edu)

^d^ Department of Agricultural Economics, Texas A&M University, 600 John Kimbrough Blvd, College Station, TX 77843. [yu.zhang@ag.tamu.edu](mailto:yu.zhang@ag.tamu.edu)

^e^ Institute for Advancing Health Through Agriculture, Texas A&M AgriLife Research, 17360 Coit Road, Dallas, TX, USA. [macmillanuribe@ag.tamu.edu](mailto:macmillanuribe@ag.tamu.edu)

Corresponding author:

Alexandra L. MacMillan Uribe

[macmillanuribe@ag.tamu.edu](mailto:macmillanuribe@ag.tamu.edu)

**Supplemental Table 1.** Missingness of Variables Used in Analysis (Eligible Participants, n = 343)

| **Group** | **Variable** | **Missing (n)** | **Missing (%)** |
| --- | --- | --- | --- |
| Categorical variables | Caregiver employment status | 114 | 33.20 |
|  | Caregiver Hispanic status | 113 | 32.90 |
|  | Caregiver gender | 111 | 32.40 |
|  | Caregiver marital status | 111 | 32.40 |
|  | Caregiver education level | 111 | 32.40 |
|  | Food shopping frequency | 0 | 0 |
|  | Food preparation frequency | 0 | 0 |
|  | Daycare frequency | 0 | 0 |
|  | Relationship to toddler | 0 | 0 |
|  | Toddler gender | 2 | 0.58 |
| Continuous variables | Number of children | 1 | 0.29 |
|  | Caregiver age (years) | 0 | 0 |
|  | Toddler age (months) | 0 | 0 |
|  | Dinner out (times per week) | 0 | 0 |
| Store preference items | Work | 54 | 15.74 |
|  | Employees | 51 | 14.87 |
|  | Ads | 51 | 14.87 |
|  | Eco | 50 | 14.58 |
|  | Aisle | 49 | 14.29 |
|  | Coupons | 49 | 14.29 |
|  | Discount | 49 | 14.29 |
|  | Clean | 47 | 13.70 |
|  | Temperature | 47 | 13.70 |
|  | Cart | 46 | 13.41 |
|  | Delivery | 46 | 13.41 |
|  | Bag | 46 | 13.41 |
|  | Sales | 46 | 13.41 |
|  | Spoil | 45 | 13.12 |
|  | Safe | 45 | 13.12 |
|  | Respond | 45 | 13.12 |
|  | Local | 44 | 12.83 |
|  | Open | 44 | 12.83 |
|  | Prices | 44 | 12.83 |
|  | Payment | 44 | 12.83 |
|  | Quick | 43 | 12.54 |
|  | Parking | 43 | 12.54 |
|  | Layout | 43 | 12.54 |
|  | Online | 43 | 12.54 |
|  | Wipes | 43 | 12.54 |
|  | Cashiers | 43 | 12.54 |
|  | Size | 42 | 12.24 |
|  | Bulk | 41 | 11.95 |
|  | Maintain | 41 | 11.95 |
|  | Organic | 40 | 11.66 |
|  | Prep | 40 | 11.66 |
|  | Variety | 39 | 11.37 |
|  | List | 39 | 11.37 |
|  | Fresh | 38 | 11.08 |
|  | Familiar | 37 | 10.79 |
|  | Other stores | 35 | 10.20 |
|  | Home | 32 | 9.33 |

**Supplemental Table 2.** Cross-classification of food shopping frequency and food preparation frequency, full sample and mothers-only subsample

| **Food preparation frequency** | **Full sample (n = 148)** | | | **Mothers-only subsample (n = 121)** | | |
| --- | --- | --- | --- | --- | --- | --- |
|  | **Shops occasionally** | **Shops sometimes** | **Shops often** | **Shops occasionally** | **Shops sometimes** | **Shops often** |
| Prepares food occasionally | 9 | 0 | 3 | 7 | 0 | 1 |
| Prepares food sometimes | 2 | 1 | 5 | 1 | 0 | 4 |
| Prepares food often | 1 | 2 | 125 | 1 | 2 | 105 |

| **Association between the two roles** | **Full sample (n = 148)** | **Mothers-only subsample (n = 121)** |
| --- | --- | --- |
| Caregivers classified the same way on both roles under the often / not-often coding (%) | 92.57 | 93.39 |
| Caregivers classified differently on the two roles under the often / not-often coding (n) | 11 | 8 |
| Phi coefficient | 0.653 | 0.633 |
| Spearman rank correlation across the three levels | 0.677 | 0.665 |

*Notes*: The table cross-classifies food shopping frequency with food preparation frequency. The agreement and discordance counts and the phi coefficient beneath the cross-classification are computed on the binary often / not-often coding of the two roles that the regression models use, while the Spearman correlation uses the three response levels. The mothers-only subsample excludes two cases with missing employment data.

**Supplemental Table 3.** Full coefficient estimates, full sample (n = 148)

| **Store-preference dimension** | **Characteristic** | **Coefficient** | **Standard error** | **95% CI lower bound** | **95% CI upper bound** | **p value** |
| --- | --- | --- | --- | --- | --- | --- |
| Store Quality and Service | Caregiver age (years) | 0.03*** | 0.01 | 0.01 | 0.05 | 0.003 |
|  | Number of children | 0.01 | 0.04 | -0.08 | 0.09 | 0.877 |
|  | Caregiver single | -0.00 | 0.12 | -0.25 | 0.24 | 0.979 |
|  | Toddler is a girl | -0.16 | 0.11 | -0.38 | 0.05 | 0.132 |
|  | Caregiver non-Hispanic Black | 0.19 | 0.15 | -0.10 | 0.48 | 0.196 |
|  | Caregiver non-Hispanic Other | 0.07 | 0.23 | -0.40 | 0.53 | 0.776 |
|  | Caregiver Hispanic | 0.32** | 0.14 | 0.04 | 0.60 | 0.027 |
|  | Caregiver high school, some college, or associate degree | 0.28 | 0.28 | -0.28 | 0.84 | 0.323 |
|  | Caregiver college degree or higher | 0.00 | 0.31 | -0.60 | 0.61 | 0.995 |
|  | Often the food shopper | -0.71*** | 0.24 | -1.19 | -0.22 | 0.004 |
|  | Often the food preparer | 0.46** | 0.22 | 0.03 | 0.89 | 0.035 |
|  | Daycare frequency | 0.02 | 0.03 | -0.05 | 0.09 | 0.551 |
|  | Dinner out (times per week) | 0.00 | 0.03 | -0.05 | 0.05 | 0.994 |
|  | SNAP participation | -0.03 | 0.13 | -0.29 | 0.22 | 0.804 |
|  | WIC participation | -0.12 | 0.11 | -0.35 | 0.10 | 0.275 |
|  | Free or reduced-price school meals | 0.02 | 0.12 | -0.22 | 0.26 | 0.857 |
|  | Father | 0.19 | 0.19 | -0.19 | 0.56 | 0.328 |
|  | Other relative | 0.06 | 0.23 | -0.41 | 0.52 | 0.814 |
| In-Store Accessibility | Caregiver age (years) | 0.02* | 0.01 | -0.00 | 0.04 | 0.071 |
|  | Number of children | 0.05 | 0.05 | -0.06 | 0.16 | 0.345 |
|  | Caregiver single | -0.11 | 0.15 | -0.41 | 0.20 | 0.490 |
|  | Toddler is a girl | -0.11 | 0.14 | -0.38 | 0.16 | 0.425 |
|  | Caregiver non-Hispanic Black | 0.16 | 0.18 | -0.21 | 0.52 | 0.401 |
|  | Caregiver non-Hispanic Other | 0.06 | 0.30 | -0.53 | 0.64 | 0.842 |
|  | Caregiver Hispanic | 0.39** | 0.18 | 0.04 | 0.74 | 0.031 |
|  | Caregiver high school, some college, or associate degree | 0.14 | 0.36 | -0.57 | 0.85 | 0.696 |
|  | Caregiver college degree or higher | 0.12 | 0.39 | -0.64 | 0.89 | 0.748 |
|  | Often the food shopper | -0.62** | 0.31 | -1.23 | -0.01 | 0.045 |
|  | Often the food preparer | 0.54** | 0.27 | 0.00 | 1.08 | 0.049 |
|  | Daycare frequency | 0.04 | 0.04 | -0.04 | 0.13 | 0.327 |
|  | Dinner out (times per week) | 0.05 | 0.03 | -0.02 | 0.11 | 0.132 |
|  | SNAP participation | -0.12 | 0.16 | -0.45 | 0.20 | 0.459 |
|  | WIC participation | -0.14 | 0.14 | -0.43 | 0.14 | 0.316 |
|  | Free or reduced-price school meals | 0.07 | 0.15 | -0.23 | 0.37 | 0.666 |
|  | Father | 0.07 | 0.24 | -0.40 | 0.54 | 0.775 |
|  | Other relative | 0.10 | 0.30 | -0.49 | 0.68 | 0.746 |
| Online and Delivery Options | Caregiver age (years) | 0.03** | 0.01 | 0.00 | 0.05 | 0.026 |
|  | Number of children | 0.05 | 0.06 | -0.06 | 0.16 | 0.389 |
|  | Caregiver single | -0.02 | 0.16 | -0.35 | 0.30 | 0.897 |
|  | Toddler is a girl | -0.28* | 0.14 | -0.56 | 0.01 | 0.057 |
|  | Caregiver non-Hispanic Black | 0.12 | 0.20 | -0.27 | 0.50 | 0.545 |
|  | Caregiver non-Hispanic Other | 0.20 | 0.31 | -0.42 | 0.82 | 0.526 |
|  | Caregiver Hispanic | 0.12 | 0.19 | -0.25 | 0.50 | 0.524 |
|  | Caregiver high school, some college, or associate degree | -0.27 | 0.38 | -1.02 | 0.48 | 0.474 |
|  | Caregiver college degree or higher | -0.29 | 0.41 | -1.10 | 0.52 | 0.482 |
|  | Often the food shopper | -0.54 | 0.33 | -1.18 | 0.11 | 0.104 |
|  | Often the food preparer | 0.30 | 0.29 | -0.27 | 0.87 | 0.298 |
|  | Daycare frequency | 0.08* | 0.04 | -0.01 | 0.17 | 0.066 |
|  | Dinner out (times per week) | 0.01 | 0.03 | -0.06 | 0.08 | 0.769 |
|  | SNAP participation | 0.18 | 0.17 | -0.16 | 0.53 | 0.292 |
|  | WIC participation | -0.03 | 0.15 | -0.34 | 0.27 | 0.820 |
|  | Free or reduced-price school meals | -0.05 | 0.16 | -0.37 | 0.27 | 0.744 |
|  | Father | 0.16 | 0.25 | -0.34 | 0.66 | 0.522 |
|  | Other relative | -0.18 | 0.31 | -0.80 | 0.44 | 0.573 |
| Price Sensitivity | Caregiver age (years) | 0.03* | 0.01 | -0.00 | 0.05 | 0.052 |
|  | Number of children | 0.04 | 0.06 | -0.08 | 0.16 | 0.493 |
|  | Caregiver single | -0.05 | 0.17 | -0.38 | 0.28 | 0.762 |
|  | Toddler is a girl | -0.26* | 0.15 | -0.55 | 0.03 | 0.082 |
|  | Caregiver non-Hispanic Black | -0.09 | 0.20 | -0.48 | 0.31 | 0.669 |
|  | Caregiver non-Hispanic Other | 0.35 | 0.32 | -0.28 | 0.98 | 0.272 |
|  | Caregiver Hispanic | 0.12 | 0.19 | -0.26 | 0.50 | 0.543 |
|  | Caregiver high school, some college, or associate degree | -0.14 | 0.39 | -0.90 | 0.62 | 0.720 |
|  | Caregiver college degree or higher | -0.52 | 0.42 | -1.35 | 0.30 | 0.213 |
|  | Often the food shopper | -0.62* | 0.33 | -1.28 | 0.04 | 0.064 |
|  | Often the food preparer | 0.84*** | 0.29 | 0.25 | 1.42 | 0.005 |
|  | Daycare frequency | 0.07 | 0.05 | -0.02 | 0.16 | 0.145 |
|  | Dinner out (times per week) | 0.06 | 0.04 | -0.01 | 0.13 | 0.113 |
|  | SNAP participation | -0.33* | 0.18 | -0.68 | 0.02 | 0.061 |
|  | WIC participation | -0.12 | 0.16 | -0.42 | 0.19 | 0.452 |
|  | Free or reduced-price school meals | 0.08 | 0.16 | -0.25 | 0.40 | 0.642 |
|  | Father | -0.21 | 0.26 | -0.72 | 0.30 | 0.421 |
|  | Other relative | -0.20 | 0.32 | -0.83 | 0.43 | 0.533 |
| Food Quality and Health | Caregiver age (years) | 0.01 | 0.01 | -0.01 | 0.03 | 0.523 |
|  | Number of children | 0.01 | 0.05 | -0.08 | 0.11 | 0.813 |
|  | Caregiver single | -0.03 | 0.14 | -0.30 | 0.24 | 0.842 |
|  | Toddler is a girl | -0.05 | 0.12 | -0.29 | 0.19 | 0.684 |
|  | Caregiver non-Hispanic Black | 0.26 | 0.16 | -0.07 | 0.58 | 0.117 |
|  | Caregiver non-Hispanic Other | 0.14 | 0.26 | -0.38 | 0.65 | 0.600 |
|  | Caregiver Hispanic | 0.31* | 0.16 | -0.01 | 0.62 | 0.054 |
|  | Caregiver high school, some college, or associate degree | 0.46 | 0.32 | -0.17 | 1.08 | 0.149 |
|  | Caregiver college degree or higher | 0.37 | 0.34 | -0.31 | 1.04 | 0.282 |
|  | Often the food shopper | -0.44 | 0.27 | -0.98 | 0.10 | 0.111 |
|  | Often the food preparer | 0.56** | 0.24 | 0.08 | 1.03 | 0.022 |
|  | Daycare frequency | 0.06* | 0.04 | -0.01 | 0.14 | 0.096 |
|  | Dinner out (times per week) | 0.01 | 0.03 | -0.05 | 0.07 | 0.744 |
|  | SNAP participation | 0.11 | 0.14 | -0.17 | 0.40 | 0.441 |
|  | WIC participation | -0.01 | 0.13 | -0.27 | 0.24 | 0.914 |
|  | Free or reduced-price school meals | 0.07 | 0.13 | -0.20 | 0.33 | 0.606 |
|  | Father | 0.27 | 0.21 | -0.15 | 0.68 | 0.210 |
|  | Other relative | -0.35 | 0.26 | -0.87 | 0.17 | 0.184 |

*Notes*: Each block reports the estimates for one store-preference dimension, with all covariates entered simultaneously. n = 148 for every model. R-squared, not adjusted R-squared, is 0.209 for store quality and service, 0.163 for in-store accessibility, 0.128 for online and delivery options, 0.213 for price sensitivity, and 0.145 for food quality and health. Reference categories are caregiver non-Hispanic White, caregiver less than high school, caregiver married or partnered, caregiver is the mother, and toddler is a boy. Stars denote *p < 0.10, **p < 0.05, and ***p < 0.01. SNAP = Supplemental Nutrition Assistance Program; WIC = Special Supplemental Nutrition Program for Women, Infants, and Children.

**Supplemental Table 4.** Full coefficient estimates, mothers-only subsample (n = 121)

| **Store-preference dimension** | **Characteristic** | **Coefficient** | **Standard error** | **95% CI lower bound** | **95% CI upper bound** | **p value** |
| --- | --- | --- | --- | --- | --- | --- |
| Store Quality and Service | Caregiver age (years) | 0.03** | 0.01 | 0.00 | 0.05 | 0.017 |
|  | Number of children | 0.00 | 0.05 | -0.10 | 0.10 | 0.979 |
|  | Caregiver single | 0.02 | 0.14 | -0.25 | 0.30 | 0.876 |
|  | Toddler is a girl | -0.15 | 0.13 | -0.40 | 0.10 | 0.244 |
|  | Caregiver non-Hispanic Black | 0.23 | 0.18 | -0.12 | 0.58 | 0.197 |
|  | Caregiver non-Hispanic Other | 0.12 | 0.27 | -0.41 | 0.65 | 0.666 |
|  | Caregiver Hispanic | 0.34** | 0.16 | 0.03 | 0.66 | 0.035 |
|  | Caregiver high school, some college, or associate degree | 0.31 | 0.33 | -0.34 | 0.97 | 0.345 |
|  | Caregiver college degree or higher | 0.07 | 0.37 | -0.66 | 0.80 | 0.852 |
|  | Often the food shopper | -0.83*** | 0.30 | -1.42 | -0.24 | 0.006 |
|  | Often the food preparer | 0.60** | 0.27 | 0.07 | 1.12 | 0.028 |
|  | Daycare frequency | 0.03 | 0.04 | -0.05 | 0.11 | 0.462 |
|  | Dinner out (times per week) | 0.00 | 0.03 | -0.07 | 0.07 | 0.993 |
|  | SNAP participation | -0.03 | 0.16 | -0.35 | 0.28 | 0.827 |
|  | WIC participation | -0.20 | 0.13 | -0.47 | 0.07 | 0.144 |
|  | Free or reduced-price school meals | -0.01 | 0.14 | -0.29 | 0.27 | 0.962 |
|  | Caregiver employed | -0.03 | 0.14 | -0.31 | 0.25 | 0.844 |
| In-Store Accessibility | Caregiver age (years) | 0.02 | 0.01 | -0.01 | 0.04 | 0.157 |
|  | Number of children | 0.04 | 0.06 | -0.07 | 0.15 | 0.449 |
|  | Caregiver single | -0.22 | 0.15 | -0.53 | 0.08 | 0.154 |
|  | Toddler is a girl | -0.04 | 0.14 | -0.32 | 0.24 | 0.793 |
|  | Caregiver non-Hispanic Black | 0.12 | 0.20 | -0.27 | 0.51 | 0.550 |
|  | Caregiver non-Hispanic Other | 0.35 | 0.30 | -0.24 | 0.94 | 0.240 |
|  | Caregiver Hispanic | 0.44** | 0.18 | 0.09 | 0.79 | 0.015 |
|  | Caregiver high school, some college, or associate degree | 0.12 | 0.37 | -0.61 | 0.85 | 0.742 |
|  | Caregiver college degree or higher | 0.16 | 0.41 | -0.66 | 0.97 | 0.704 |
|  | Often the food shopper | -0.75** | 0.33 | -1.40 | -0.09 | 0.025 |
|  | Often the food preparer | 0.68** | 0.30 | 0.09 | 1.26 | 0.025 |
|  | Daycare frequency | 0.07 | 0.05 | -0.02 | 0.16 | 0.119 |
|  | Dinner out (times per week) | 0.06 | 0.04 | -0.01 | 0.14 | 0.103 |
|  | SNAP participation | -0.10 | 0.18 | -0.44 | 0.25 | 0.580 |
|  | WIC participation | -0.18 | 0.15 | -0.48 | 0.11 | 0.226 |
|  | Free or reduced-price school meals | -0.04 | 0.16 | -0.35 | 0.27 | 0.805 |
|  | Caregiver employed | -0.23 | 0.16 | -0.54 | 0.08 | 0.149 |
| Online and Delivery Options | Caregiver age (years) | 0.03* | 0.01 | -0.00 | 0.06 | 0.052 |
|  | Number of children | 0.03 | 0.06 | -0.10 | 0.15 | 0.685 |
|  | Caregiver single | -0.08 | 0.17 | -0.42 | 0.26 | 0.637 |
|  | Toddler is a girl | -0.18 | 0.16 | -0.50 | 0.13 | 0.249 |
|  | Caregiver non-Hispanic Black | 0.27 | 0.22 | -0.16 | 0.71 | 0.217 |
|  | Caregiver non-Hispanic Other | 0.37 | 0.33 | -0.29 | 1.03 | 0.268 |
|  | Caregiver Hispanic | 0.18 | 0.20 | -0.21 | 0.57 | 0.367 |
|  | Caregiver high school, some college, or associate degree | -0.34 | 0.41 | -1.16 | 0.48 | 0.414 |
|  | Caregiver college degree or higher | -0.36 | 0.46 | -1.27 | 0.55 | 0.437 |
|  | Often the food shopper | -0.79** | 0.37 | -1.51 | -0.06 | 0.035 |
|  | Often the food preparer | 0.81** | 0.33 | 0.15 | 1.47 | 0.016 |
|  | Daycare frequency | 0.06 | 0.05 | -0.04 | 0.17 | 0.215 |
|  | Dinner out (times per week) | 0.02 | 0.04 | -0.07 | 0.10 | 0.690 |
|  | SNAP participation | 0.18 | 0.20 | -0.21 | 0.57 | 0.360 |
|  | WIC participation | -0.01 | 0.17 | -0.35 | 0.32 | 0.937 |
|  | Free or reduced-price school meals | -0.14 | 0.18 | -0.49 | 0.20 | 0.413 |
|  | Caregiver employed | 0.08 | 0.17 | -0.27 | 0.43 | 0.642 |
| Price Sensitivity | Caregiver age (years) | 0.02 | 0.01 | -0.01 | 0.05 | 0.116 |
|  | Number of children | 0.01 | 0.06 | -0.12 | 0.14 | 0.865 |
|  | Caregiver single | -0.07 | 0.18 | -0.42 | 0.28 | 0.680 |
|  | Toddler is a girl | -0.04 | 0.16 | -0.36 | 0.28 | 0.808 |
|  | Caregiver non-Hispanic Black | -0.03 | 0.23 | -0.48 | 0.42 | 0.887 |
|  | Caregiver non-Hispanic Other | 0.51 | 0.34 | -0.17 | 1.18 | 0.139 |
|  | Caregiver Hispanic | 0.23 | 0.20 | -0.17 | 0.63 | 0.263 |
|  | Caregiver high school, some college, or associate degree | -0.01 | 0.42 | -0.84 | 0.83 | 0.990 |
|  | Caregiver college degree or higher | -0.15 | 0.47 | -1.08 | 0.78 | 0.752 |
|  | Often the food shopper | -0.53 | 0.38 | -1.28 | 0.21 | 0.159 |
|  | Often the food preparer | 0.84** | 0.34 | 0.17 | 1.51 | 0.015 |
|  | Daycare frequency | 0.10* | 0.05 | -0.00 | 0.21 | 0.058 |
|  | Dinner out (times per week) | 0.03 | 0.04 | -0.05 | 0.12 | 0.435 |
|  | SNAP participation | -0.35* | 0.20 | -0.75 | 0.05 | 0.083 |
|  | WIC participation | -0.17 | 0.17 | -0.51 | 0.17 | 0.328 |
|  | Free or reduced-price school meals | -0.12 | 0.18 | -0.47 | 0.24 | 0.504 |
|  | Caregiver employed | -0.33* | 0.18 | -0.68 | 0.03 | 0.069 |
| Food Quality and Health | Caregiver age (years) | 0.01 | 0.01 | -0.01 | 0.04 | 0.303 |
|  | Number of children | 0.01 | 0.05 | -0.09 | 0.12 | 0.835 |
|  | Caregiver single | -0.14 | 0.15 | -0.43 | 0.15 | 0.353 |
|  | Toddler is a girl | -0.07 | 0.14 | -0.34 | 0.20 | 0.616 |
|  | Caregiver non-Hispanic Black | 0.53*** | 0.19 | 0.16 | 0.90 | 0.006 |
|  | Caregiver non-Hispanic Other | 0.46 | 0.28 | -0.10 | 1.02 | 0.108 |
|  | Caregiver Hispanic | 0.46*** | 0.17 | 0.12 | 0.80 | 0.008 |
|  | Caregiver high school, some college, or associate degree | 0.21 | 0.35 | -0.49 | 0.91 | 0.549 |
|  | Caregiver college degree or higher | 0.18 | 0.39 | -0.60 | 0.96 | 0.646 |
|  | Often the food shopper | -0.54* | 0.31 | -1.16 | 0.09 | 0.091 |
|  | Often the food preparer | 0.74** | 0.28 | 0.18 | 1.30 | 0.010 |
|  | Daycare frequency | 0.04 | 0.04 | -0.05 | 0.13 | 0.382 |
|  | Dinner out (times per week) | 0.06* | 0.04 | -0.01 | 0.14 | 0.093 |
|  | SNAP participation | 0.06 | 0.17 | -0.27 | 0.39 | 0.713 |
|  | WIC participation | 0.00 | 0.14 | -0.28 | 0.29 | 0.988 |
|  | Free or reduced-price school meals | 0.03 | 0.15 | -0.26 | 0.33 | 0.832 |
|  | Caregiver employed | -0.03 | 0.15 | -0.32 | 0.27 | 0.859 |

*Notes:* Each block reports the estimates for one store-preference dimension, with all covariates entered simultaneously. n = 121 for every model. R-squared, not adjusted R-squared, is 0.222 for store quality and service, 0.254 for in-store accessibility, 0.153 for online and delivery options, 0.219 for price sensitivity, and 0.203 for food quality and health. Reference categories are caregiver non-Hispanic White, caregiver less than high school, caregiver married or partnered, caregiver not employed, and toddler is a boy. Stars denote *p < 0.10, **p < 0.05, and ***p < 0.01. SNAP = Supplemental Nutrition Assistance Program; WIC = Special Supplemental Nutrition Program for Women, Infants, and Children.

**Supplemental Table 5.** Participation in food and nutrition assistance programs for full sample and mothers-only subsample

| **Program** | **Full sample (n = 148)** | | **Mothers-only subsample (n = 121)** | |
| --- | --- | --- | --- | --- |
|  | **Count** | **%** | **Count** | **%** |
| SNAP | 111 | 75.00 | 91 | 75.21 |
| WIC | 58 | 39.19 | 47 | 38.84 |
| Head Start / Early Head Start | 11 | 7.43 | 9 | 7.44 |
| Free or reduced-price school meals | 47 | 31.76 | 38 | 31.40 |
| School fresh fruit and vegetable program | 13 | 8.78 | 9 | 7.44 |
| School summer food service program | 9 | 6.08 | 6 | 4.96 |
| Commodity supplemental food program | 5 | 3.38 | 3 | 2.48 |
| The emergency food assistance program | 8 | 5.41 | 7 | 5.79 |
| Participated in 1 program | 78 | 52.70 | 62 | 51.24 |
| Participated in 2 programs | 47 | 31.76 | 41 | 33.88 |
| Participated in 3 programs | 14 | 9.46 | 12 | 9.92 |
| Participated in 4 or more programs | 9 | 6.08 | 6 | 4.96 |

*Notes*: SNAP = Supplemental Nutrition Assistance Program; WIC = Special Supplemental Nutrition Program for Women, Infants, and Children. Percentages may not total 100% due to rounding.

**Supplemental Table 6.** Caregiver race and Hispanic status as reported, full sample (n = 148) and mothers-only subsample (n = 121)

| **Characteristic** | **Group** | **Full sample (n = 148)** | | **Mothers-only subsample (n = 121)** | |
| --- | --- | --- | --- | --- | --- |
|  |  | **Count** | **%** | **Count** | **%** |
| Caregiver race | White | 79 | 53.38 | 61 | 50.41 |
|  | Black | 44 | 29.73 | 37 | 30.58 |
|  | Other | 20 | 13.51 | 18 | 14.88 |
|  | Asian | 5 | 3.38 | 5 | 4.13 |
| Caregiver Hispanic status | Not Hispanic | 103 | 69.59 | 78 | 64.46 |
|  | Hispanic | 45 | 30.41 | 43 | 35.54 |

*Notes*: Rows report caregiver race and caregiver Hispanic status as reported by respondents. The four mutually exclusive race and ethnicity categories used in the regression models, which are shown in Table 2, combine these two responses, with non-Hispanic Asian and non-Hispanic Other respondents combined into a single non-Hispanic Other category because of small cell sizes. Percentages may not total 100% due to rounding. The mothers-only subsample excludes two cases with missing employment data.

**Supplemental Table 7.** Bivariate comparisons of store-preference dimensions by subgroup, full sample (n = 148)

| **Subgroup characteristic** | **Groups (n)** | **Store-preference dimension** | **F** | **Numerator df** | **Denominator df** | **p value** |
| --- | --- | --- | --- | --- | --- | --- |
| Toddler gender | Girl 80; Boy 68 | Factor 1: Store Quality and Service | 0.98 | 1 | 146 | 0.325 |
|  |  | Factor 2: In-Store Accessibility | 0.32 | 1 | 146 | 0.572 |
|  |  | Factor 3: Online and Delivery Options | 2.97 | 1 | 146 | 0.087 |
|  |  | Factor 4: Price Sensitivity | 2.25 | 1 | 146 | 0.136 |
|  |  | Factor 5: Food Quality and Health | 0.02 | 1 | 146 | 0.878 |
| Caregiver race | White 79; Black 44; Other 20; Asian 5 | Factor 1: Store Quality and Service | 1.10 | 3 | 144 | 0.351 |
|  |  | Factor 2: In-Store Accessibility | 0.88 | 3 | 144 | 0.453 |
|  |  | Factor 3: Online and Delivery Options | 0.41 | 3 | 144 | 0.744 |
|  |  | Factor 4: Price Sensitivity | 2.19 | 3 | 144 | 0.092 |
|  |  | Factor 5: Food Quality and Health | 0.60 | 3 | 144 | 0.617 |
| Caregiver Hispanic status | Not Hispanic 103; Hispanic 45 | Factor 1: Store Quality and Service | 3.84 | 1 | 146 | 0.052 |
|  |  | Factor 2: In-Store Accessibility | 5.50 | 1 | 146 | 0.020 |
|  |  | Factor 3: Online and Delivery Options | 0.07 | 1 | 146 | 0.785 |
|  |  | Factor 4: Price Sensitivity | 2.51 | 1 | 146 | 0.116 |
|  |  | Factor 5: Food Quality and Health | 2.23 | 1 | 146 | 0.138 |
| Caregiver marital status | Married/Partnered 94; Single 54 | Factor 1: Store Quality and Service | 0.01 | 1 | 146 | 0.936 |
|  |  | Factor 2: In-Store Accessibility | 1.32 | 1 | 146 | 0.253 |
|  |  | Factor 3: Online and Delivery Options | 0.03 | 1 | 146 | 0.871 |
|  |  | Factor 4: Price Sensitivity | 0.02 | 1 | 146 | 0.877 |
|  |  | Factor 5: Food Quality and Health | 0.05 | 1 | 146 | 0.819 |
| Caregiver education level | Less than high school 6; High school 114; College and higher 28 | Factor 1: Store Quality and Service | 2.14 | 2 | 145 | 0.121 |
|  |  | Factor 2: In-Store Accessibility | 0.29 | 2 | 145 | 0.747 |
|  |  | Factor 3: Online and Delivery Options | 0.20 | 2 | 145 | 0.819 |
|  |  | Factor 4: Price Sensitivity | 1.06 | 2 | 145 | 0.350 |
|  |  | Factor 5: Food Quality and Health | 1.83 | 2 | 145 | 0.164 |
| Food preparation frequency | Occasionally 12; Sometimes 8; Often 128 | Factor 1: Store Quality and Service | 0.54 | 2 | 145 | 0.586 |
|  |  | Factor 2: In-Store Accessibility | 2.41 | 2 | 145 | 0.093 |
|  |  | Factor 3: Online and Delivery Options | 0.11 | 2 | 145 | 0.900 |
|  |  | Factor 4: Price Sensitivity | 3.75 | 2 | 145 | 0.026 |
|  |  | Factor 5: Food Quality and Health | 1.46 | 2 | 145 | 0.237 |

*Notes*: Each block reports a one-way analysis of variance comparing mean factor scores across the groups of one characteristic. Cell counts, means, and standard deviations are group-specific, while the F statistic, its degrees of freedom, and the p value are common to the groups of a characteristic within a dimension.

**Supplemental Table 8.** Household food roles entered as a single combined variable, three specifications

| **Sample** | **Store-preference dimension** | **Specification** | **Coefficient** | **Standard error** | **95% CI lower bound** | **95% CI upper bound** | **p value** | **Wald p,**  **both role coefficients**  **jointly zero** | **Wald p,**  **the two role coefficients**  **equal** | **Wald p,**  **the two role coefficients**  **equal and opposite** |
| --- | --- | --- | --- | --- | --- | --- | --- | --- | --- | --- |
| Full sample (n = 148) | Store Quality and Service | Both roles often (single indicator) | -0.02 | 0.16 | -0.33 | 0.29 | 0.907 | 0.017 | 0.006 | 0.204 |
|  |  | Count of roles performed often (0, 1, or 2) | -0.08 | 0.10 | -0.27 | 0.11 | 0.417 | 0.017 | 0.006 | 0.204 |
|  |  | Net both-versus-neither contrast | -0.24 | 0.19 | -0.62 | 0.13 | 0.204 | 0.017 | 0.006 | 0.204 |
|  | In-Store Accessibility | Both roles often (single indicator) | 0.06 | 0.20 | -0.33 | 0.45 | 0.750 | 0.092 | 0.029 | 0.741 |
|  |  | Count of roles performed often (0, 1, or 2) | 0.00 | 0.12 | -0.24 | 0.24 | 0.979 | 0.092 | 0.029 | 0.741 |
|  |  | Net both-versus-neither contrast | -0.08 | 0.24 | -0.56 | 0.40 | 0.741 | 0.092 | 0.029 | 0.741 |
|  | Online and Delivery Options | Both roles often (single indicator) | -0.13 | 0.21 | -0.54 | 0.28 | 0.526 | 0.265 | 0.138 | 0.366 |
|  |  | Count of roles performed often (0, 1, or 2) | -0.09 | 0.13 | -0.34 | 0.17 | 0.504 | 0.265 | 0.138 | 0.366 |
|  |  | Net both-versus-neither contrast | -0.23 | 0.26 | -0.74 | 0.28 | 0.366 | 0.265 | 0.138 | 0.366 |
|  | Price Sensitivity | Both roles often (single indicator) | 0.29 | 0.21 | -0.13 | 0.71 | 0.179 | 0.020 | 0.012 | 0.413 |
|  |  | Count of roles performed often (0, 1, or 2) | 0.16 | 0.13 | -0.10 | 0.42 | 0.222 | 0.020 | 0.012 | 0.413 |
|  |  | Net both-versus-neither contrast | 0.22 | 0.26 | -0.30 | 0.73 | 0.413 | 0.020 | 0.012 | 0.413 |
|  | Food Quality and Health | Both roles often (single indicator) | 0.22 | 0.17 | -0.13 | 0.56 | 0.215 | 0.072 | 0.035 | 0.576 |
|  |  | Count of roles performed often (0, 1, or 2) | 0.10 | 0.11 | -0.11 | 0.31 | 0.366 | 0.072 | 0.035 | 0.576 |
|  |  | Net both-versus-neither contrast | 0.12 | 0.21 | -0.30 | 0.54 | 0.576 | 0.072 | 0.035 | 0.576 |
| Mothers-only subsample (n = 121) | Store Quality and Service | Both roles often (single indicator) | 0.01 | 0.20 | -0.38 | 0.40 | 0.948 | 0.020 | 0.006 | 0.323 |
|  |  | Count of roles performed often (0, 1, or 2) | -0.07 | 0.12 | -0.32 | 0.17 | 0.546 | 0.020 | 0.006 | 0.323 |
|  |  | Net both-versus-neither contrast | -0.24 | 0.24 | -0.71 | 0.24 | 0.323 | 0.020 | 0.006 | 0.323 |
|  | In-Store Accessibility | Both roles often (single indicator) | 0.09 | 0.22 | -0.34 | 0.52 | 0.684 | 0.048 | 0.014 | 0.788 |
|  |  | Count of roles performed often (0, 1, or 2) | 0.01 | 0.13 | -0.26 | 0.28 | 0.948 | 0.048 | 0.014 | 0.788 |
|  |  | Net both-versus-neither contrast | -0.07 | 0.26 | -0.60 | 0.45 | 0.788 | 0.048 | 0.014 | 0.788 |
|  | Online and Delivery Options | Both roles often (single indicator) | 0.15 | 0.24 | -0.33 | 0.63 | 0.532 | 0.042 | 0.013 | 0.925 |
|  |  | Count of roles performed often (0, 1, or 2) | 0.06 | 0.15 | -0.23 | 0.36 | 0.672 | 0.042 | 0.013 | 0.925 |
|  |  | Net both-versus-neither contrast | 0.03 | 0.30 | -0.56 | 0.61 | 0.925 | 0.042 | 0.013 | 0.925 |
|  | Price Sensitivity | Both roles often (single indicator) | 0.30 | 0.24 | -0.19 | 0.78 | 0.228 | 0.049 | 0.037 | 0.311 |
|  |  | Count of roles performed often (0, 1, or 2) | 0.20 | 0.15 | -0.11 | 0.50 | 0.199 | 0.049 | 0.037 | 0.311 |
|  |  | Net both-versus-neither contrast | 0.31 | 0.30 | -0.29 | 0.91 | 0.311 | 0.049 | 0.037 | 0.311 |
|  | Food Quality and Health | Both roles often (single indicator) | 0.31 | 0.20 | -0.10 | 0.71 | 0.133 | 0.036 | 0.020 | 0.417 |
|  |  | Count of roles performed often (0, 1, or 2) | 0.14 | 0.13 | -0.11 | 0.40 | 0.266 | 0.036 | 0.020 | 0.417 |
|  |  | Net both-versus-neither contrast | 0.21 | 0.25 | -0.29 | 0.71 | 0.417 | 0.036 | 0.020 | 0.417 |

*Notes*: Each row replaces the two separate role indicators with a single combined measure in the model for that dimension, holding all other covariates as specified in the main models. The net both-versus-neither contrast and the Wald test that the two role coefficients are equal and opposite are the same restriction written two ways, so those two columns report one result rather than two. The three Wald columns refer to the retained two-variable model and are therefore common to the three specification rows within a dimension and sample. No specification produces a role coefficient significant at p < 0.10 in any of the ten models.
